# Senescence atlas reveals an aged-like inflamed niche that blunts muscle regeneration

**DOI:** 10.1038/s41586-022-05535-x

**Published:** 2022-12-21

**Authors:** Victoria Moiseeva, Andrés Cisneros, Valentina Sica, Oleg Deryagin, Yiwei Lai, Sascha Jung, Eva Andrés, Juan An, Jessica Segalés, Laura Ortet, Vera Lukesova, Giacomo Volpe, Alberto Benguria, Ana Dopazo, Salvador Aznar Benitah, Yasuteru Urano, Antonio del Sol, Miguel A. Esteban, Yasuyuki Ohkawa, Antonio L. Serrano, Eusebio Perdiguero, Pura Muñoz-Cánoves

**Affiliations:** 1 Department of Medicine and Life Sciences, Pompeu Fabra University, Barcelona, Spain; 2 CIBERNED, Barcelona, Spain; 3 Laboratory of Integrative Biology, Guangzhou Institutes of Biomedicine and Health, Chinese Academy of Sciences, Guangzhou, China; 4 Key Laboratory of Regenerative Biology, Guangzhou Institutes of Biomedicine and Health, Chinese Academy of Sciences, Guangzhou, China; 5 Guangdong Provincial Key Laboratory of Stem Cells and Regenerative Medicine, Guangzhou Institutes of Biomedicine and Health, Chinese Academy of Sciences, Guangzhou, China; 6 CIC bioGUNE-BRTA (Basque Research and Technology Alliance), Bizkaia Technology Park, Derio, Spain; 7 University of Science and Technology of China, Hefei, China; 8 Genomic Unit, Centro Nacional de Investigaciones Cardiovasculares and CIBERCV, Madrid, Spain; 9 ICREA, Barcelona, Spain; 10 Institute for Research in Biomedicine and BIST, Barcelona, Spain; 11 Laboratory of Chemistry & Biology, Graduate School of Pharmaceutical Sciences and School of Medicine, The University of Tokyo, Tokyo, Japan; 12 Computational Biology Group, Luxembourg Centre for Systems Biomedicine, University of Luxembourg, Esch-sur-Alzette, Luxembourg; 13 IKERBASQUE, Basque Foundation for Science, Bilbao, Spain; 14 Bioland Laboratory, Guangzhou Regenerative Medicine and Health Guangdong Laboratory, Guangzhou, China; 15 Division of Transcriptomics. Medical Institute of Bioregulation, Kyushu University, Fukuoka, Japan; 16 Altos labs Inc, San Diego, CA USA; 17 Cardiovascular Regeneration Program, CNIC Centro Nacional de Investigaciones Cardiovasculares, Madrid, Spain

**Keywords:** Senescence, Regeneration

## Abstract

Tissue regeneration requires coordination between resident stem cells and local niche cells^1,2^. Here we identify that senescent cells are integral components of the skeletal muscle regenerative niche that repress regeneration at all stages of life. The technical limitation of senescent-cell scarcity^3^ was overcome by combining single-cell transcriptomics and a senescent-cell enrichment sorting protocol. We identified and isolated different senescent cell types from damaged muscles of young and old mice. Deeper transcriptome, chromatin and pathway analyses revealed conservation of cell identity traits as well as two universal senescence hallmarks (inflammation and fibrosis) across cell type, regeneration time and ageing. Senescent cells create an aged-like inflamed niche that mirrors inflammation associated with ageing (inflammageing^4^) and arrests stem cell proliferation and regeneration. Reducing the burden of senescent cells, or reducing their inflammatory secretome through CD36 neutralization, accelerates regeneration in young and old mice. By contrast, transplantation of senescent cells delays regeneration. Our results provide a technique for isolating in vivo senescent cells, define a senescence blueprint for muscle, and uncover unproductive functional interactions between senescent cells and stem cells in regenerative niches that can be overcome. As senescent cells also accumulate in human muscles, our findings open potential paths for improving muscle repair throughout life.

## Abstract (web summary)

A lifetime cartography of in vivo senescent cells shows that they are heterogeneous. Senescent cells create an aged-like inflamed niche that mirrors inflammation associated with ageing and arrests stem cell proliferation and tissue regeneration.

## Main

Tissue regeneration is necessary for life. Its success requires coordinated communication between resident stem cells and the surrounding niche cells. Examples can be found in the crosstalk between skeletal muscle stem cells (called satellite cells (SCs)) and macrophages or fibroadipogenic progenitors (FAPs; also called mesenchymal cells) (reviewed in refs. ^1,2^). However, the identities of the niche cellular components and secreted factors that regulate tissue regeneration are not fully characterized. During ageing, tissue regenerative functions decline, in part due to stem cell-intrinsic accumulation of damage (for example, DNA damage, loss of proteostasis and oxidative damage), functional decay of niche cells and increased inflammation (inflammageing)^4–9^ through uncharacterized mechanisms.

Cellular senescence is a state of durable cell-cycle arrest of dysfunctional cells, which acquire a bioactive senescence-associated secretory phenotype (SASP)^10^. Senescent cells accumulate with ageing and age-associated diseases, limiting lifespan and healthspan in mice^11–16^. These cells also accumulate after irradiation and impair muscle function^17^. By contrast, senescent cells have shown beneficial effects as tumour suppressors, during embryo development, and in liver and skin repair^18–21^ or reprogramming^22–24^. However, little is known about the identity of senescent cells in vivo or their contribution to tissue regeneration owing to their scarcity and presumed heterogeneity, and the lack of universal senescence markers and isolation methods^3^.

Here we isolated and defined senescent cells in vivo at the molecular and functional levels. We demonstrate that senescent cells are integral regenerative niche components that repress regeneration at all stages of life. Using single-cell transcriptomics, a method to separate senescent from non-senescent subsets of various niche cell types, and deep transcriptomics and chromatin analyses, we produced a lifetime atlas of in vivo senescent cells in regenerating skeletal muscle.

## The injured niche gains senescent cells

**Fig. 1:**
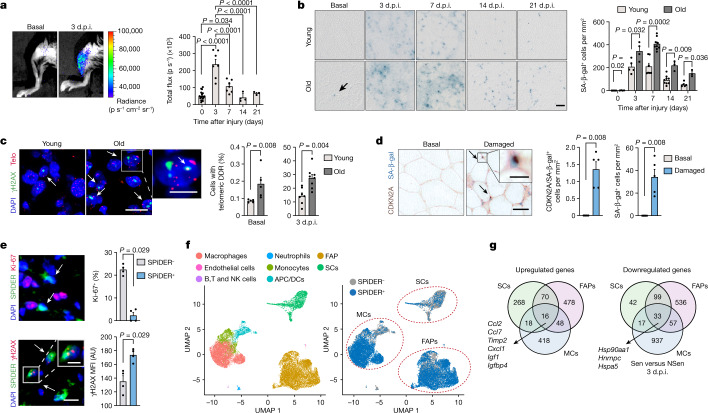
Senescent cells in regenerating muscle of young and old mice. **a**–**e**, Representative images and quantification. **a**, Luciferase activity in the muscles of young (3–6 months) p16-3MR mice at the indicated d.p.i. with CTX. p s^−1^, photons per second. *n* = 18 muscles from 12 mice (basal), 7 muscles from 5 mice (3 d.p.i.), 7 muscles from 6 mice (7 d.p.i.) and 4 muscles from 3 mice (14 d.p.i. and 21 d.p.i.). **b**, SA-β-gal^+^ cells in the TA of young mice (*n* = 5 (0, 3 and 21 d.p.i.), *n* = 7 (7 d.p.i.) and *n* = 9 (14 d.p.i.) mice) or old mice (aged more than 28 months; *n* = 4 (0 and 3 d.p.i.), *n* = 9 (7 d.p.i.) and *n* = 3 (14 and 21 d.p.i.) mice). **c**, cells with telomeric DDR (telo) in basal and 3 d.p.i. TA from young mice (*n* = 5 (basal) and *n* = 7 (3 d.p.i.) mice) and old mice (*n* = 5 (basal) and *n* = 8 (3 d.p.i.) mice). **d**, SA-β-gal and CDKN2A^+^ immunohistochemistry of uninjured or damaged human muscle samples (*n* = 5 samples per group; aged 81 ± 7.5 years). The arrows show double-positive cells. **e**, Ki-67 positivity (*n* = 4 mice per group) and γH2AX intensity (*n* = 4 mice per group) in SPiDER^+^ and SPiDER^−^ cells in 4 d.p.i. TA. AU, arbitrary units; MFI, mean fluorescence intensity. **f**, Complete 21,449-cell transcriptomic atlas from SPiDER^+^ and SPiDER^−^ samples of 3 d.p.i. young muscles (right). Data are shown as a uniform manifold approximation and projection (UMAP) to visualize variation in single-cell transcriptomes. Unsupervised clustering resolved at least eight cell types (colour coded). Left, SPiDER^+^ cells ascribed to main populations. APC, antigen-presenting cell; DCs, dendritic cells; NK, natural killer. **g**, The overlap between differentially expressed genes in SCs, FAPs and MCs at 3 d.p.i. from scRNA-seq (Sen versus NSen, false-discovery rate (FDR) < 0.05). Scale bars, 50 μm (**b** and **d**, low magnification), 10 μm (**c** and **e**, low magnification) and 5 μm (**c**–**e**, high magnification). For **a**–**e**, data are mean ± s.e.m. *P* values were calculated using Tukey’s tests (**a**) and Mann–Whitney *U*-tests (**b**–**e**). Source data

**Extended Data Fig. 1:**
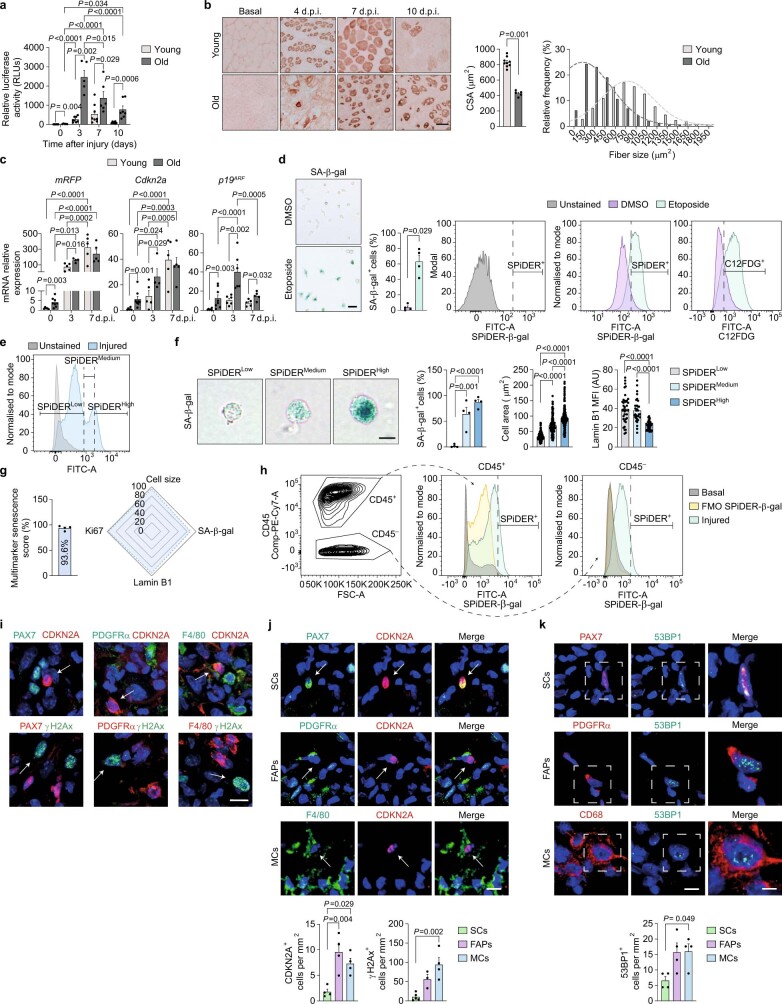
Characteristics of senescent cells in regenerating muscle of young and old mice. **a**) Quantification of Renilla luciferase activity in regenerating muscle from young and old mice at the indicated d.p.i. The luciferase activities are normalized to the activity of basal young muscle (*n* = 6 mice for young basal, 8 muscles from 6 mice for young 3 d.p.i., 8 muscles from 4 mice for young 7 d.p.i., 7 muscles from 5 mice for young 10 d.p.i., 5 mice for old basal, 5 muscles from 4 mice for old 3 d.p.i., 6 muscles from 4 mice for old 7 d.p.i., and 7 muscles from 4 mice for old 10 d.p.i.). **b**) Representative images and quantification of CSA and frequency distribution analysis of MYH3^+^ fibre size in TA muscles from young and old mice at 7 d.p.i. (*n* = 9 TA from 6 mice for young and 5 mice for old). **c**) RT-qPCR of *mRFP, Cdkn2a*, and *p19*^*ARF*^ in young and old muscle tissue from p16-3MR mice at the indicated d.p.i. (*n* = 4 mice in *mRFP* and *Cdkn2a* for old 3 d.p.i., 5 TA from 3 mice for old 7 d.p.i., 5 TA from 4 mice for young 7 d.p.i., 5 mice in *mRFP*, for young basal and 3 d.p.i. and *Cdkn2a* for young 3 d.p.i., 6 TA from 5 mice in *p19*^*ARF*^ for old 3 d.p.i., 6 mice in *Cdkn2a* and *p19*^*ARF*^ for young basal and *p19*^*ARF*^ for young 3 d.p.i., and 8 mice for old basal). **d**) Freshly isolated SCs were obtained from skeletal muscle tissue and cultured in the presence of etoposide (1 μM) or DMSO for 4 days. Cells were stained with SPiDER, C12FDG, or SA-β-gal staining in parallel and analysed by flow cytometry or microscopy to assess their entry into senescence (*n* = 4 mice/group). Unstained samples were used to determine the threshold for C12FDG and SPiDER populations. Histogram representation of SPiDER intensity, representative images of SA-β-gal and quantification are shown. **e**) Histogram representation of SPiDER-β-gal staining and gating strategy employed for isolation of SPiDER^Low^, SPiDER^Medium^, and SPiDER^High^ populations from injured skeletal muscle at 3 d.p.i. **f**) Representative images and quantification of SA-β-gal staining (*n* = 4 mice/group), cell area (*n* = 205 SPiDER^Low^ cells, 168 SPiDER^Medium^ cells, and 220 SPiDER^High^ cells), and lamin B1 expression (*n* = 36 SPiDER^Low^ cells, 33 SPiDER^Medium^ and SPiDER^High^ cells) in freshly sorted SPiDER^Low^, SPiDER^Medium^, and SPiDER^High^ from regenerating muscles at 3 d.p.i. **g**) Graphs representing multimarker senescence scoring in freshly sorted SPiDER^+^ population from 3 d.p.i. regenerating tissue of young mice. Scoring was calculated with cell size, SA-β-gal, lamin B1, and proliferation rate (Fig. 1e and Extended Data Fig. 1f; see Methods). **h**) Gating strategy used to isolate SPiDER^+^ cells from regenerating muscle tissue of young mice at 3 d.p.i. Cells were divided into two major populations with anti-CD45 antibodies to overcome differences in auto-fluorescence of hematopoietic and non-hematopoietic populations. Fluorescence Minus One (FMO) and samples from non-injured muscle tissue were used to set the threshold for SPiDER^+^ staining within each cell population. **i**) Representative immunofluorescence images showing CDKN2A^+^ and γH2Ax^+^ cells in proximity to SCs, FAPs and MCs identified with anti-Pax7, anti-PDGFRα and anti-F4/80 antibodies, respectively, in regenerating muscles of young mice at 4 d.p.i.. Arrows indicate CDKN2A^+^ and γH2Ax^+^ cells. **j**) Representative images and quantification of CDKN2A^+^ cells (*n* = 4 mice/group) and γH2Ax^+^ cells (*n* = 5 mice for SCs, 3 mice for FAPs, and 4 mice for MCs) in regenerating muscles from young mice at 4 d.p.i. Each cell type was labelled with indicated antibodies and nuclei with 4,6-diaminido-2-phenylindole (DAPI). Arrows indicate CDKN2A^+^ cells. **k**) Representative images and quantification of 53BP1^+^ cells in regenerating human muscle. Each cell type was labelled with indicated antibodies, and nuclei with DAPI (*n* = 4 samples/group, from persons aged 81±7.5 years old). Scale bars: 50 μm (**b**); 10 μm (**d**, **f**, **i**, **j**, and **k** low magnification) and 5 μm (**k** high magnification). Results are displayed as means±s.e.m; *P* values were calculated by Mann–Whitney *U*-test (**a** between ages, **c** between ages, **b** and **d**), and Tukey’s test (**a** between d.p.i., **c** between d.p.i., **f**, **j**, and **k**). Source data

To study cellular senescence in skeletal muscle, we used p16-3MR mice, which express *Renilla* luciferase, monomeric red fluorescent protein (RFP) and a viral thymidine kinase under the *Cdkn2a* (also known as *p16*^*INK4a*^) promoter^18^, and analysed luciferase activity (a proxy of CDKN2A-expressing senescent cells). We also used wild-type (WT) mice and analysed senescence-associated beta-galactosidase (SA-β-gal) activity as a marker of senescent cells. We induced injury by intramuscular injection of cardiotoxin (CTX) into either young mice (aged 3–6 months) or very old mice (28-month-old mice and older (geriatric age)). Neither luciferase nor SA-β-gal activities were detectable in resting muscles, but were substantially induced at 3 days post-injury (d.p.i.) and were still elevated at 7 d.p.i., after which they decreased (Fig. 1a,b and Extended Data Fig. 1a). Senescent cells were more abundant and persisted longer in injured muscles of old mice (Fig. 1b and Extended Data Fig. 1a), correlating with their less efficient recovery process^5,6^ (Extended Data Fig. 1b). Moreover, the telomeric DNA-damage response (DDR) (γH2AX telomere immunostaining) was also higher in regenerating muscle from old compared with from young mice or in non-injured (basal) muscle (Fig. 1c). Additional markers confirmed that injury induced a transient accumulation of senescent cells in both age groups (Extended Data Fig. 1c). Similar to in mouse muscle, SA-β-gal- and CDKN2A-positive cells were present in biopsy samples of damaged adult human muscles (Fig. 1d). Thus, senescent cells are induced in the damaged muscle of both humans and mice.

To determine the nature of senescent cells in vivo, we established a fluorescence-activated cell sorting (FACS) method based on the fluorescent probe SPiDER-β-gal (hereafter, SPiDER; similar to the C12FDG fluorescent probe)^25,26^, which labels all SA-β-gal^+^ cells (Extended Data Fig. 1d). No cells were labelled by SPiDER in unperturbed muscle. By contrast, in injured muscles, cells with a high SPiDER signal (SPiDER^+^) and SPiDER^−^ cells were detected (Extended Data Fig. 1e). The SPiDER^+^ fraction contained cells that were highly enriched for all of the tested senescence markers (such as SA-β-gal activity, larger cell size, lamin B1 reduction, cell-cycle arrest and increased DNA damage) (Fig. 1e and Extended Data Fig. 1f). Scoring for four of these markers, the SPiDER^+^ cell fraction was 93.6% enriched in senescent cells, demonstrating the specificity of the separation method (Extended Data Fig. 1g).

We next aimed to generate a single-cell RNA-sequencing (scRNA-seq) atlas of senescent cells from regenerating muscle in an unbiased manner. To control for cell-type differences in autofluorescence, and to obtain potential minor, non-haematopoietic senescent cells, we sorted SPiDER^+^ and SPiDER^−^ fractions from CD45^+^ and CD45^−^ populations and merged them after scRNA-seq (Extended Data Fig. 1h). The SPiDER^+^ populations comprised predominantly monocytes and macrophages (hereafter, myeloid cells (MCs)), FAPs, and SCs and their progeny, while smaller populations included antigen-presenting cells, neutrophils, endothelial cells, and B, T, and natural killer cells (Fig. 1f). Differential expression analysis revealed a core signature of 16 upregulated and 33 downregulated genes in the three major senescent populations (Fig. 1g), of which the former included inflammatory and matrix-remodelling/fibrotic SASP factors (such as *Ccl2*, *Ccl7*, *Igfbp4* and *Timp2*).

Immunostaining of senescence markers (CDKN2A and γH2AX) labelled cells close to the three major niche cell types identified by scRNA-seq (for example, PAX7^+^ for SCs; PDGFRα^+^ for FAPs and F4/80^+^ for MCs) (Extended Data Fig. 1i) in regenerating mouse tissue. Furthermore, markers for SCs, FAPs and MCs co-localized with senescence markers (in the order MCs ≥ FAPs >  SCs) (Extended Data Fig. 1j). Importantly, damaged human muscle also contained SCs (PAX7^+^), FAPs (PDGFRα^+^) and MCs (CD68^+^) positive for the DDR marker 53BP1 (Extended Data Fig. 1k). Together, these results provide: (1) a strategy to isolate senescent cells from tissue that does not rely on transgenic mice; (2) a single-cell cartography of senescent cells in vivo; and (3) the identity of new senescent-cell niche constituents after injury.

## Senescence hampers muscle regeneration

**Fig. 2:**
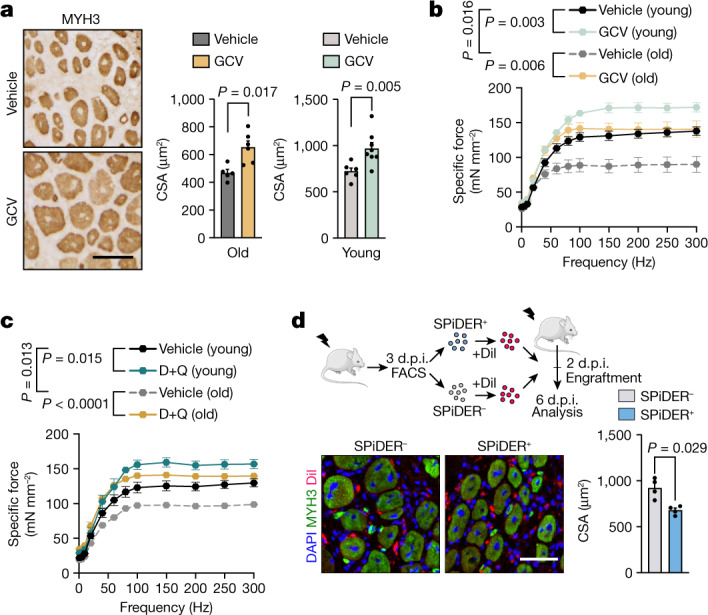
Cellular senescence hampers muscle regeneration throughout life. **a**, Representative images and quantification of the cross-sectional area (CSA) of embryonic myosin heavy chain (MYH3)-positive fibres in regenerating TA from vehicle- or GCV-treated old (*n* = 5 TA from 3 mice (vehicle) and *n* = 6 TA from 3 mice (GCV)) and young (*n* = 6 TA from 4 mice (vehicle) and *n* = 8 TA from 4 mice (GCV)) p16-3MR mice at 7 d.p.i. **b**, Specific force–frequency curves of EDL muscles of vehicle- or GCV-treated young (*n* = 11 EDL from 7 mice (vehicle) and *n* = 9 EDL from 7 mice (GCV)) and old (*n* = 5 EDL from 4 mice (vehicle) and *n* = 7 EDL from 5 mice (GCV)) p16-3MR mice at 10 d.p.i. **c**, Specific force–frequency curves as described in **b**, but for vehicle- and D+Q-treated young (*n* = 8 EDL from 5 mice (vehicle) and *n* = 7 EDL from 5 mice (D+Q)) and old (*n* = 5 EDL from 3 mice (vehicle) and *n* = 7 EDL from 5 mice (D+Q group)) mice at 10 d.p.i. **d**, An equal number of SPiDER^+^ or SPiDER^−^ cells from young 3 d.p.i. regenerating muscles were stained with Dil and transplanted into the preinjured TA of young recipient mice for 4 days. *n* = 4 mice per group. Strategy schematic, representative images and quantification of the CSA of MYH3^+^ fibres are shown. The schematic in **d** was created using Servier Medical Art, CC BY 3.0. Scale bars, 50 μm (**a** and **d**). For **a**–**d**, data are mean ± s.e.m. *P* values were calculated using two-way analysis of variance (ANOVA) and mixed-effects analysis (**b** and **c**) and Mann–Whitney *U*-tests (**a** and **d**). Source data

**Extended Data Fig. 2:**
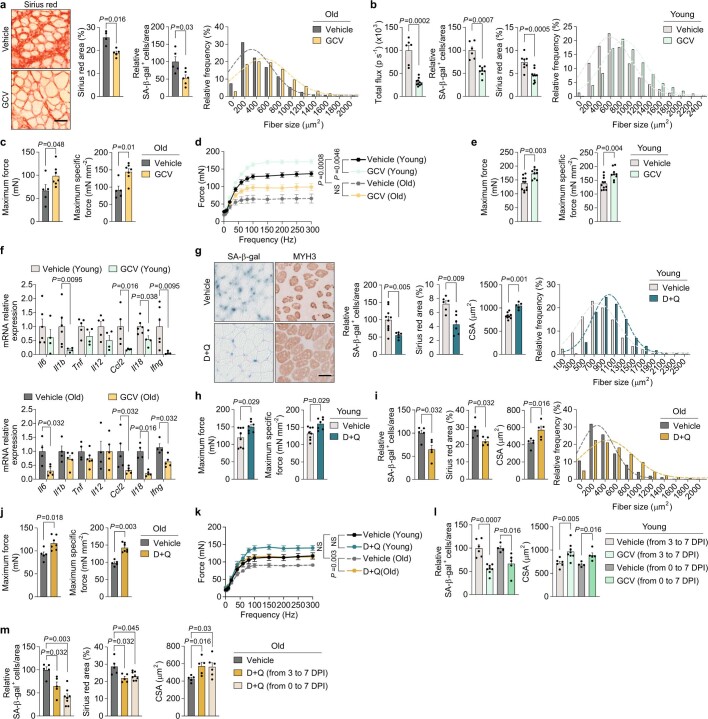
Removal of senescent cells improves skeletal muscle regeneration after acute damage throughout life. Young and old p16-3MR mice were subjected to CTX injury, treated with vehicle or GCV during the course of regeneration and analysed at 7 d.p.i. **a**) Representative images of Sirius Red staining and quantification of SA-β-gal^+^ cells in damaged area (*n* = 5 TA from 4 mice for vehicle and 6 TA from 3 mice for GCV), Sirius Red staining (*n* = 4 TA from 3 mice for vehicle and 5 TA from 3 mice for GCV), and frequency distribution analysis of positive MYH3 fibre size (*n* = 3 mice/group) in cryosections from old p16-3MR mice. **b**) Quantification of *in vivo* Renilla luminescence activity (*n* = 6 TA from 3 mice for vehicle and 10 TA from 5 mice for GCV), SA-β-gal^+^ cells (*n* = 6 TA from 4 mice for vehicle and 8 TA from 4 mice for GCV), Sirius Red staining (n = 8 TA from 5 mice for vehicle and 10 mice from 5 mice for GCV), and frequency distribution analysis of positive MYH3 fibre size (*n* = 4 mice/group) in cryosections from vehicle- and GCV-treated young p16-3MR mice. **c**) Force measurements in EDL muscles of vehicle- or GCV-treated old p16-3MR mice at 10 d.p.i. (*n* = 5 EDL muscles from 4 mice for vehicle and 7 EDL from 5 mice for GCV). **d**) Force-frequency curves measured in EDL muscles of vehicle- or GCV-treated young (*n* = 11 EDL from 7 mice in vehicle and 9 EDL from 7 mice in GCV group) and old (*n* = 5 EDL from 4 mice in vehicle and 7 EDL from 5 mice in GCV group) p16-3MR mice at 10 d.p.i. **e**) Force measurements in EDL muscles of vehicle- or GCV-treated young p16-3MR mice at 10 d.p.i. (*n* = 11 EDL muscles from 8 mice for vehicle and 9 EDL from 7 mice for GCV). **f**) RT-qPCR of *Il6*, *Il1b*, *Tnf*, *Il12*, *Ccl2*, *Il18*, and *Ifng* in muscle tissue from young (top, *n* = 5 TA muscles in *Tnf*, *Il12* and *Ccl2*, and 6 TA muscles in *Il6*, *Il1b*, *Il18* and *Ifng* from 4 mice for vehicle and 4 mice for GCV) and old (bottom, *n* = 4 TA muscles from 3 mice for vehicle and 6 TA muscles in *Tnf* and 5 TA in *Il6*, *Il1b*, *Il12*, *Ccl2*, *Il18*, *Ifng* from 3 mice for GCV) p16-3MR mice. **g**) Muscles of young WT mice were injured with CTX and treated with vehicle or senolytics during regeneration and analysed at 7 d.p.i. Representative images, SA-β-gal staining (*n* = 10 TA from 5 mice for vehicle and 6 TA from 3 mice for D+Q), mean CSA (*n* = 9 TA from 6 mice for vehicle and 5 TA from 3 mice for D+Q) and frequency distribution analysis (*n* = 3 mice/group) of MYH3^+^ fibres, and Sirius Red quantification (*n* = 6 TA from 3 mice for both groups) in TA cryosections from young WT mice. **h**) Force measurements in EDL muscles of vehicle- and D+Q-treated young mice at 10 d.p.i. (*n* = 8 EDL from 5 mice in vehicle and 7 EDL from 5 mice in D+Q group). **i**) As in **g**, SA-β-gal staining, mean CSA and frequency distribution analysis of MYH3^+^ fibres and Sirius Red quantification in TA cryosections from old WT mice (*n* = 5 mice/group) . **j**) As in **h**, force measurements in EDL muscles of vehicle- and D+Q-treated old mice at 10 d.p.i. (*n* = 5 EDL from 3 mice in vehicle and 7 EDL from 5 mice in D+Q group). **k**) Force-frequency curves measured in EDL muscles of vehicle- and D+Q-treated young (*n* = 8 EDL from 5 mice in vehicle and 7 EDL from 5 mice in D+Q group), and old (*n* = 5 EDL from 3 mice in vehicle and 7 EDL from 5 mice in D+Q group) mice at 10 d.p.i. **l**) SA-β-gal^+^ cells and mean CSA in vehicle- or GCV-treated young p16-3MR mice after 4-days or 7-days treatment at 7 d.p.i. (*n* = 6 muscles from 4 mice for vehicle (3 to 7), 8 muscles from 4 mice GCV (3 to 7), 4 mice for vehicle (0 to 7), and 5 mice for GCV (0 to 7)). **m**) Quantification of SA-β-gal, Sirius Red staining (*n* = 5 mice for vehicle and D+Q (3 to 7) and 8 muscles from 4 mice for D+Q (0 to 7)), and mean CSA of MYH3^+^ fibres (*n* = 5 mice for vehicle and D+Q (3 to 7) and 6 muscles from 3 mice for D+Q (0 to 7)) in vehicle- and D+Q- treated old mice after 4-days or 7-days treatment at 7 d.p.i. (*n* = 5–8 muscles from 4-5 mice). Treatments were administered from 3 to 7 d.p.i. in **b**, **g**, and **i**, from 0 to 7 d.p.i. in **a**, **b**, and **f** and from 3 to 10 d.p.i. in **c**, **d**, **e**, **h**, **j** and **k**. Scale bars 50 μm. Results are displayed as mean ± s.e.m.; *P* values were calculated by two-way ANOVA and Mixed-effects analysis (**d** and **k**) and Mann–Whitney *U*-test (**a**, **b**, **c**, **e**, **f**, **g**, **h**, **i**, **j**, **l** and **m**). Source data

To determine the role of senescent cells in muscle regeneration, we treated young and old p16-3MR mice daily with ganciclovir (GCV) during this process. GCV reduced the presence of senescent cells in injured muscle, indexed by lower luciferase activity and number of SA-β-gal^+^cells (Extended Data Fig. 2a, b). GCV treatment rescued defective muscle regeneration, reduced inflammation and fibrosis, and enhanced force generation in old mice, and accelerated the regenerative process in young p16-3MR mice (Fig. 2a,b and Extended Data Fig. 2c–f). Daily treatment with the senolytic compounds dasatinib and quercetin (hereafter D+Q)^15^ also reduced SA-β-gal^+^ cell number, accelerated regeneration, increased force, and reduced matrix deposition and inflammation at both ages (Fig. 2c and Extended Data Fig. 2g–k). Similar benefits were obtained when GCV or D+Q were transiently administered (starting at 3 d.p.i.) (Extended Data Fig. 2l,m). Consistently, transplantation of sorted SPiDER^+^ (senescent) and SPiDER^−^ (non-senescent) cells, labelled with the lipophilic vital dye Dil, into preinjured muscle revealed that only SPiDER^+^ cells delayed regeneration of young host muscles (Fig. 2d). Thus, senescent cells were detrimental to muscle regeneration in both young and old mice.

## Senescence in acute and chronic damage

**Extended Data Fig. 3:**
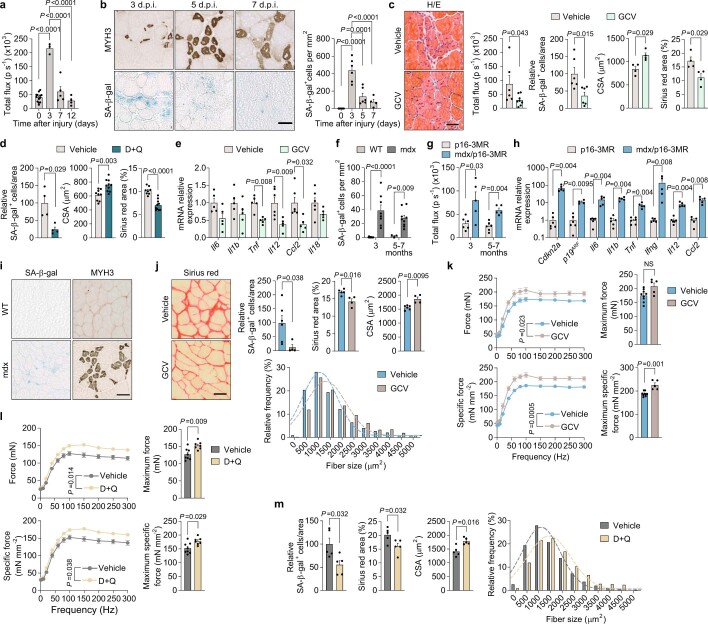
Removal of senescent cells improves skeletal muscle regeneration after microdamage and after chronic damage. **a**) Quantification of *in vivo* Renilla luciferase activity in young p16-3MR mice at the indicated days after injury by micropunctures (*n* = 13 mice for Basal, 3 mice for 3 d.p.i., 5 mice for 7 d.p.i., and 4 mice for 12 d.p.i.). **b**) Representative images of SA-β-gal and MYH3 staining in cryosections of TA muscles from young WT mice at the indicated days after injury by micropunctures and quantification of SA-β-gal are shown (*n* = 5 mice for basal, 6 mice for 3 d.p.i. and 5 d.p.i., and 4 mice for 7 d.p.i.). **c**) Young p16-3MR mice were subjected to micropunctures in TA muscles and treated for 7 days with GCV, starting from the day of injury, sacrificed at 7 d.p.i. and TA muscles were analysed. Representative images of hematoxylin and eosin (H/E) staining and quantification of *in vivo* Renilla luminescence activity in TA (*n* = 6 TA from 4 mice for vehicle and 8 TA from 4 mice for GCV), SA-β-gal^+^ cells (*n* = 6 TA from 4 mice for both groups), CSA of centrally nucleated fibres and Sirius Red staining (*n* = 4 mice/group). **d**) Young WT mice were subjected to micropunctures injury in TA muscles and treated for 7 days with D+Q, starting from the day of injury. Quantification of SA-β-gal^+^ cells (*n* = 4 mice/group), CSA of centrally nucleated fibres in cryosections (*n* = 9 TA from 5 mice for vehicle and 10 TA from 5 mice for D+Q), Sirius Red staining (*n* = 8 TA from 4 mice for vehicle and 10 TA from 5 mice for D+Q). **e**) As in **c**, mRNA quantification of the indicated genes by RT-qPCR in TA muscles (*n* = 5 mice in *Il6*, *Il1b*, and *Tnf* and 6 mice in, *Il12, Ccl2*, and *Il18* for vehicle and 5 TA from 4 mice for GCV). **f**) Quantification of SA-β-gal^+^ cells in uninjured TA muscles from WT and mdx mice at indicated age (*n* = 9 mice WT^3months^ and mdx^5–7months^, 3 mice WT^5–7months^, and 8 mice mdx^3months^). **g**) *In vivo* quantification of Renilla luminescence activity in basal muscles of p16-3MR and mdx/p16-3MR mice at indicated age (*n* = 6 mice p16-3MR^3months^ and p16-3MR^5–7months^ and 5 muscles from 3 mice mdx^3months^ and mdx^5–7months^). **h**) mRNA quantification of the indicated genes by RT-qPCR in TA muscles from young WT and mdx/p16-3MR mice of 5 months of age (*n* = 5 mice in *Ifng* and *Ccl2* and 6 mice for the rest of the genes for WT and 4 mice in *p19*^*ARF*^ and 5 mice for the rest of the genes for mdx/p16-3MR). **i**) Representative images of SA-β-gal and MYH3 staining in cryosections of TA muscles from young WT and mdx mice. **j**) Young mdx/p16-3MR mice received GCV twice a week for 2 months and were sacrificed at 5 months of age. Representative images are shown for Sirius Red staining as well as for quantification of SA-β-gal^+^ cells, Sirius Red staining, CSA and frequency distribution of regenerating fibres in TA muscles of vehicle- or GCV-treated mdx/p16-3MR mice (*n* = 6 mice for vehicle and 4 mice for GCV). **k**) Young mdx/p16-3MR mice were treated with GCV of vehicle for 2 months and force measurements were performed in EDL muscles at 5 months of age. Graphs represent maximum and specific force parameters and force-frequency curves (*n* = 9 EDL from 6 mice for vehicle and 5 EDL from 3 mice for GCV). **l**) Young mdx mice received D+Q twice a week for 2 months and muscle samples were collected at 5 months of age. Maximum and specific force parameters and force-frequency curves in EDL muscles are represented (*n* = 8 EDL muscles from 5 mice for vehicle and 7 EDL from 6 mice for D+Q). **m**) Quantifications of SA-β-gal^+^ cells, mean CSA and frequency distribution of regenerating fibres and Sirius Red staining in muscle cryosections (*n* = 5 mice/group) of vehicle- and D+Q-treated mdx mice after 2 months of treatment. Scale bars 50 μm. Results are displayed as mean ± s.e.m.; *P* values were calculated by Tukey’s test (**a** and **b**), two-way ANOVA and Mixed-effects analysis in (**k** and **l** force-frequency curves) and Mann–Whitney *U*-test (**c**, **d**, **e**, **f**, **g**, **h**, **j**, **k**, **l** and **m**). Source data

To exclude that the observed roles of senescent cells depended on the degree of tissue injury, we (1) induced transient, mild injury with localized muscle micropunctures; and (2) examined the chronic, severely injured muscle of mdx mice (a model of Duchenne muscular dystrophy)^27^. Muscle micropunctures induced acute and transient SA-β-gal^+^ cell presence and luminescence activity in WT and p16-3MR mice, respectively (Extended Data Fig. 3a,b). Reducing the accumulation of senescent cells in p16-3MR mice (by GCV) or WT mice (by D+Q) increased the size of regenerating myofibres and decreased inflammation and matrix deposition (Extended Data Fig. 3c–e). Next, p16-3MR mice were brought to the mdx background. Senescent cells were more numerous and persistent in the muscles of mdx/p16-3MR mice compared with in the non-dystrophic muscles of p16-3MR mice (Extended Data Fig. 3f–h), in agreement with chronic damage in mdx muscles (Extended Data Fig. 3i). Longer-term treatment (twice weekly for 2 months) of mdx/p16-3MR mice with GCV or mdx mice with D+Q reduced the burden of senescent cells and alleviated the regenerative impairment, as shown by larger myofibres, lower fibrosis and enhanced muscle force (Extended Data Fig. 3j–m). Thus, irruption of senescent cells in the muscle niche, either transiently (in mild injury) or persistently (in chronic injury), was deleterious for regeneration, irrespective of age. These findings challenge the prevalent view that senescent cells are beneficial when transiently present after acute injury, particularly in young tissue (reviewed in refs. ^28,29^).

## Heterogeneity of senescent cells in vivo

**Extended Data Fig. 4:**
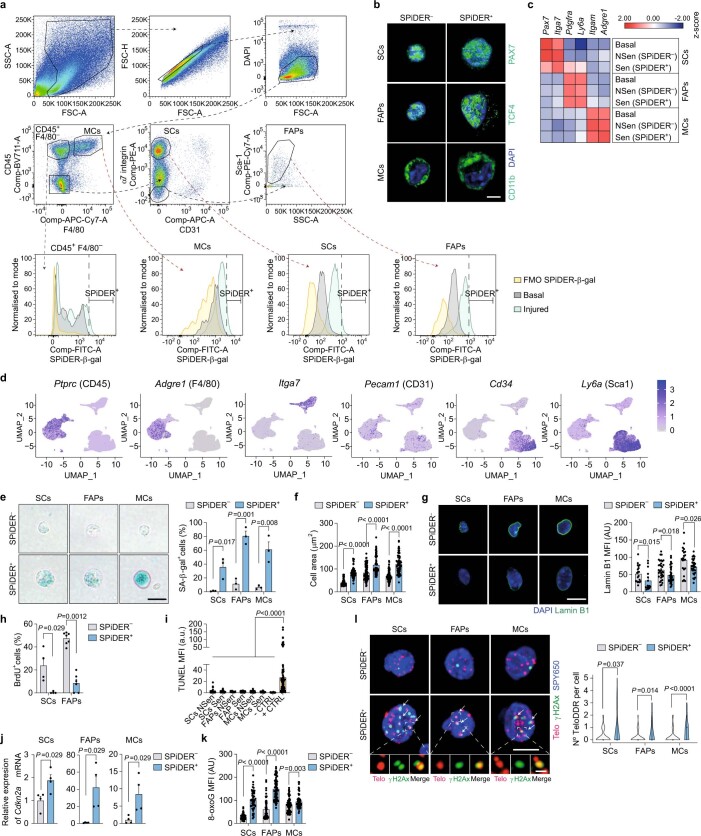
Isolation and characterization of different senescent cell types. **a**) Gating strategy used to simultaneously isolate SCs, FAPs, and MCs from WT mice. Representative histogram plots from cytofluorimetric analysis are employed to assess SPiDER levels in the cell populations. FMO controls and non-injured samples were used to determine the threshold for SPiDER within each cell population. **b**) Representative pictures of Pax7, TCF4 and CD11b expression in sorted SCs, FAPs and MCs respectively. **c**) Heatmap of gene expression levels of the indicated genes in basal, NSen and Sen SCs, FAPs and MCs. **d**) Single-cell expression levels for select gene markers. **e**) Representative images and quantification of SA-β-gal staining of freshly sorted SPiDER^+^ and SPiDER^–^ SCs, FAPs, and MCs from regenerating muscles at 3 d.p.i. (*n* = 3 mice/group). **f**) As in **e**, quantification of the cell area (*n* = 79 SCs^NSen^, 55 SCs^Sen^, 94 FAPs^NSen^, 75 FAPs^Sen^, 106 MCs^NSen^, and 66 MCs^Sen^). **g**) Representative images and quantification of lamin B1 expression of freshly sorted SPiDER^+^ and SPiDER^–^ SCs, FAPs, and MCs from regenerating muscles at 3 d.p.i., (*n* = 20 SCs^NSen^, 15 SCs^Sen^, 33 FAPs^NSen^, 35 FAPs^Sen^, 21 MCs^NSen^, and 30 MCs^Sen^; arbitrary units: AU). **h**) BrdU incorporation was quantified in cells obtained at 7 d.p.i. and cultured for 3 days (*n* = 4 mice in SCs^NSen^ and SCs^Sen^, 7 mice in FAPs^NSen^, and 6 mice in FAPs^Sen^). **i**) Quantification of TUNEL assay in freshly sorted SPiDER^+^ and SPiDER^–^ SCs, FAPs and MCs. Cells treated with DNase were used as a positive control (*n* = 64 SCs^NSen^, 63 SCs^Sen^, 55 FAPs^NSen^, 73 FAPs^Sen^, 67 MCs^NSen^, 56 MCs^Sen^, 32 neg. control and 101 pos. control cells). **j**) RT-qPCR of *Cdkn2a* in freshly sorted SPiDER^+^ and SPiDER^–^ SCs, FAPs, and MCs from regenerating muscles at 3 d.p.i. (*n* = 4 mice/group). **k**) As in **e**, quantification of 8-oxoG (*n* = 47 SCs^NSen^, 49 SCs^Sen^, 60 FAPs^NSen^, 74 FAPs^Sen^, 65 MCs^NSen^, and 65 MCs^Sen^). **l**) Representative images and quantification of telomeric DDR in SPiDER^+^ and SPiDER^–^ sorted cells from regenerating muscle of young mice at 3 d.p.i. (*n* = 53 SCs^NSen^, 64 SCs^Sen^, 45 FAPs^NSen^, 47 FAPs^Sen^, 46 MCs^NSen^, and 45 MCs^Sen^). Scale bars, 10 μm (**e** and **g**), 5 μm (**l** low magnification), and 1 μm (**b** and **l** high magnification). Results are displayed as mean ± s.e.m.; *P* values from multiple *t*-tests (**e**), Tukey’s test (**i**), and Mann–Whitney *U*-test (**f**, **g**, **h**, **j**, **k** and **l**). Source data

For a deeper characterization of the three main populations of senescent cells from regenerating muscle, we established a FACS method based on the coupling of cell-surface markers of SCs, FAPs and MCs to the SPiDER reagent (Extended Data Fig. 4a). The method provided simultaneous isolation of the SPiDER^+^ and SPiDER^−^ subpopulations (hereafter referred to as senescent (Sen) and non-senescent (NSen) cells) of the three cell types, as validated by immunostaining and gene expression (Extended Data Fig. 4b,c), and at single-cell level (Extended Data Fig. 4d). Each Sen subpopulation scored positively for six accepted senescence markers—namely, increased SA-β-gal activity, large cell size, decreased proliferation, reduced lamin B1, lack of apoptotic programmed death and high *Cdkn2a* expression (Extended Data Fig. 4e–j). Moreover, both 8-oxoguanine (an indicator of reactive oxygen species (ROS)-induced DNA damage) (Extended Data Fig. 4k) and telomeric DDR were increased in the Sen subpopulations (Extended Data Fig. 4l), confirming the high level of senescent-cell enrichment of the SPiDER-sorting protocol.

**Extended Data Fig. 5:**
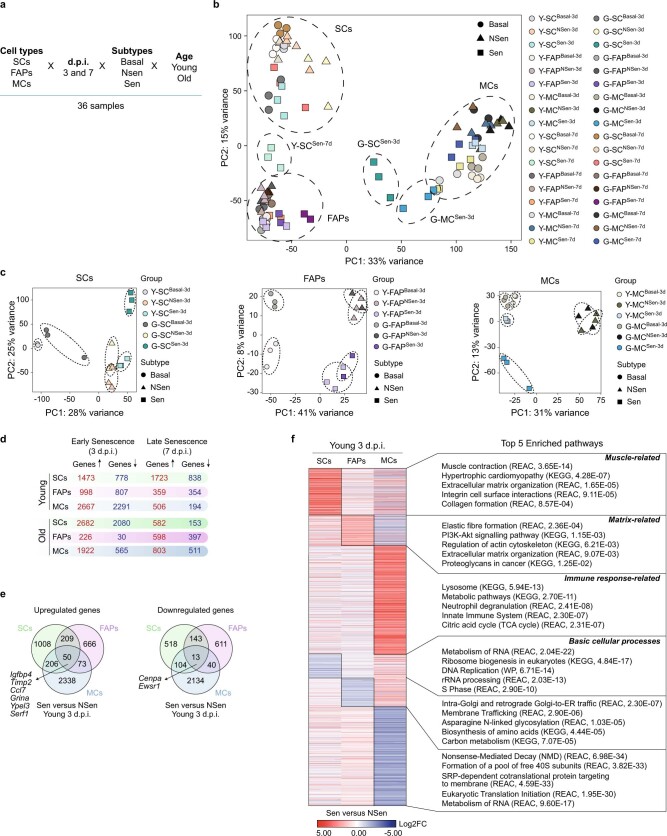
RNAseq analysis of the different senescent cell types throughout life. **a**) Scheme showing 36 different conditions (3 cell types x 12 conditions) assessed by RNA-seq. **b**) Principal component analysis (PCA) of the full transcriptome of senescent (Sen), non-senescent (NSen), and Basal SCs, FAPs, and MCs isolated from resting (Basal) and regenerating muscles of young and old mice at 3 and 7 d.p.i. **c**) PCA of Sen, NSen, and Basal SCs, FAPs, and MCs from basal and regenerating muscles at 3 d.p.i. of young and old mice. **d**) Scheme indicating the number of differentially expressed genes in Sen vs NSen SCs, FAPs, and MCs from young and old mice at 3 and 7 d.p.i. (FDR <0.05). **e**) Venn-diagram showing the overlap between differentially expressed genes in SCs, FAPs, and MCs from young mice at 3 d.p.i. (Sen vs NSen were compared, FDR <0.05). **f**) Heatmap of genes that were differentially expressed (DE) uniquely by one population of interest and the corresponding canonical pathways enrichment (CP) analysis (g:Profiler web server). The heatmap shows log_2_FC for Sen versus (vs) their NSen counterparts isolated from regenerating muscles of young mice at 3 d.p.i. Source data

For in-depth transcriptomic analysis of the distinct cell populations, we performed bulk RNA-seq analysis of Sen and NSen SCs, FAPs and MCs at early (3 d.p.i.) and late (7 d.p.i.) phases of regeneration, and NSen cells from basal (contralateral) muscles from young or old mice (a total of 36 distinct in vivo conditions) (Extended Data Fig. 5a). Principal component analysis (PCA) of all of the datasets revealed that the transcriptomes primarily clustered by cell type, rather than cell state, regeneration timepoint or mouse age (Extended Data Fig. 5b). Within each cell type, there was clear segregation according to cell state, with a strong influence of senescence (Extended Data Fig. 5c).

**Extended Data Fig. 6:**
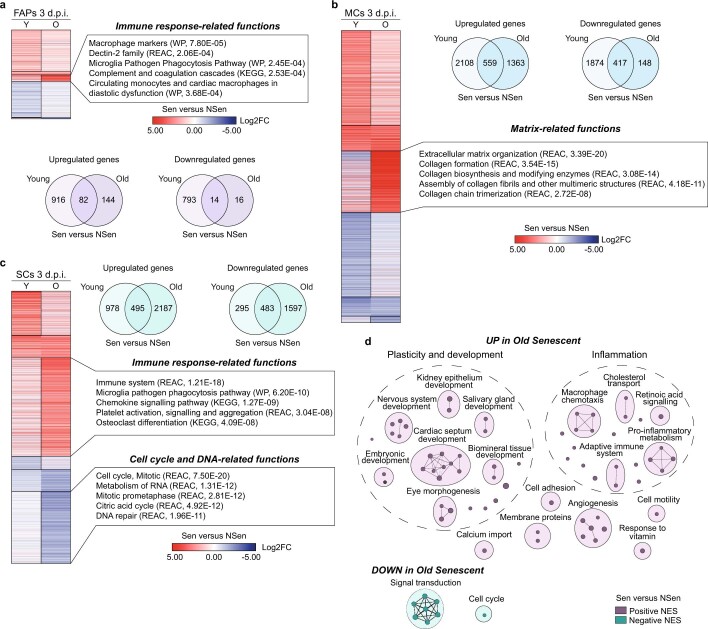
Further RNAseq analysis of the different senescent cell types. **a**) (top) Heatmap of unique and common differentially expressed genes in young (Y) or old (O) Sen FAPs and CP enrichment (g:Profiler web server) of exclusively differentially expressed genes in G conditions. (bottom) Venn-diagram showing the overlap between differentially expressed genes of Sen FAPs from young and old mice at 3 d.p.i. **b**) As in **a** for MCs. **c**) As in **a** for SCs. **d**) Clusters of gene sets (GSEA) differentially enriched at 3 d.p.i. in old Sen populations, but not in young Sen populations. Gene sets were considered common with FDR < 0.25 for all 3 old Sen populations with the exclusion of gene sets common for at least 2 young Sen populations. Node size is proportional to the number of genes identified in each gene set. Grey edges indicate gene overlap. Source data

A comparison between the Sen and NSen subpopulations at 3 d.p.i. in young mice showed 2,251 differentially expressed genes in Sen SCs, 1,805 in Sen FAPs and 4,958 in Sen MCs with little overlap (Extended Data Fig. 5d,e). Many differentially expressed genes and enriched pathways were specific to each Sen cell type and revealed the cell of origin: muscle contraction and integrin/cell-surface interactions in Sen SCs; actin cytoskeleton and elastic-fibre regulation in Sen FAPs; and immune functions and high lysosomal content in MCs (Extended Data Fig. 5f). Basic cell processes were downregulated in each Sen subpopulation (Extended Data Fig. 5f). However, with ageing, Sen cells gained alternative cell-fate traits, including macrophage-specific pathways and complement/coagulation cascades in Sen FAPs (Extended Data Fig. 6a); extracellular matrix organization and collagen formation in Sen MCs (Extended Data Fig. 6b); and immune features in Sen SCs (Extended Data Fig. 6c). All Sen cells from old mice gained further cell plasticity and pro-inflammatory traits (Extended Data Fig. 6d), which, in addition to their higher number (Fig. 1b,c and Extended Data Fig. 1a,c), may contribute to muscle regenerative failure at very old age (Extended Data Fig. 1b).

## Tissue injury and ageing drive senescence

**Fig. 3:**
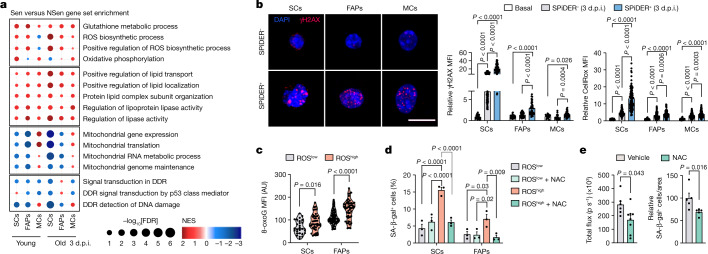
Tissue injury and ageing prime niche cells for senescence through oxidative stress and DNA damage. **a**, Common upregulated and downregulated gene sets (gene set enrichment analysis (GSEA), FDR < 0.25) related to the indicated functions in Sen versus NSen SCs, FAPs and MCs from young and old mice at 3 d.p.i. **b**, Representative images of γH2AX and quantification of γH2AX (*n* = 24 SCs (basal), 91 SCs (NSen), 78 SCs (Sen), 26 FAPs (basal), 34 FAPs (NSen), 35 FAPs (Sen), 20 MCs (basal), 22 MCs (NSen) and 29 MCs (Sen)) and CellRox levels (*n* = 24 SCs (basal), 95 SCs (NSen), 98 SCs (Sen), 26 FAPs (basal), 81 FAPs (NSen), 104 FAPs (Sen), 20 MCs (basal), 93 MCs (NSen) and 97 MCs (Sen)) in sorted SC, FAP and MC populations from basal and regenerating muscles at 3 d.p.i. **c**, Quantification of 8-oxoguanine (8-oxoG) levels in sorted ROS^high^ and ROS^low^ SCs and FAPs from young muscle at 1 d.p.i. (*n* = 27 SCs (ROS^low^), *n* = 40 SCs (ROS^high^), *n* = 66 FAPs (ROS^low^) and *n* = 50 FAPs (ROS^high^)). **d**, ROS^high^ and ROS^low^ SCs and FAPs were isolated from regenerating muscle at 1 d.p.i., and were cultured in vitro for 3 days with or without NAC. Quantification of SA-β-gal^+^ cells in each population compared with basal cells. *n* = 3 mice per group. **e**, Young p16-3MR mice were injured with CTX and treated with NAC during regeneration. Left, *Renilla* luciferase activity in the TA at 4 d.p.i. *n* = 6 TA muscles from 6 mice (vehicle) and *n* = 8 TA muscles from 4 mice (NAC group). Right, quantification of SA-β-gal^+^ cells. *n* = 5 TA muscles from 4 mice in each group. For **b**, scale bar, 10 μm. For **b**, **d** and **e**, data are mean ± s.e.m. *P* values were calculated using Tukey’s tests (**d**) and Mann–Whitney *U*-tests (**b**, **c** and **e**). Source data

**Extended Data Fig. 7:**
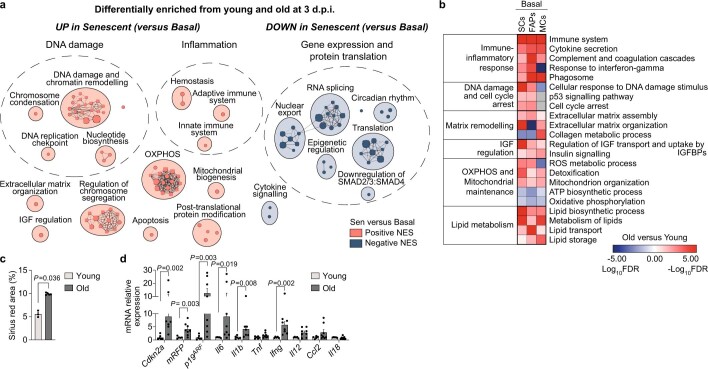
Comparison of the senescence and basal transcriptomes, and of the basal transcriptomes throughout life. Fibrosis and inflammation inold muscles. **a**) Clusters of gene sets (GSEA) differentially enriched from Sen vs Basal SCs, FAPs, and MCs from young and old mice at 3 d.p.i. Gene sets were considered common with FDR < 0.25 in at least 5/6 comparisons. Node size is proportional to the number of genes identified in each gene set. Grey edges indicate gene overlap. **b**) Heatmap of gene sets enriched in DE genes from Old vs Young SCs, FAPs, and MCs (g:Profiler web server, FDR < 0.05) isolated from non-injured muscle tissue. **c**) Quantification of Sirius Red in TA muscles from young and old mice (*n* = 3 mice in young and 5 mice in old). **d**) mRNA quantification by RT-qPCR of indicated genes in TA muscles from young and old mice (*n* = 6 mice in *Cdkn2a*, *p19*^*ARF*^, *Il1b* and *Tnf* and 5 mice in the rest of genes for young group, 7 mice in *Cdkn2a* and *Il1b* and 8 mice in the rest of genes for old group). Results are displayed as mean ± s.e.m.; *P* values were calculated using a Mann–Whitney *U*-test (**c** and **d**). Source data

To understand how senescence is induced in the regenerative niche, we searched for pathways that are commonly enriched in all Sen cells early after injury (3 d.p.i.). Compared with basal cells, DNA-damage, cell-cycle regulation and inflammation pathways were upregulated, whereas homeostatic gene expression and protein translation pathways were downregulated in Sen cells (Extended Data Fig. 7a). Compared with NSen cells, Sen cells were enriched in pathways that are implicated in cellular stress, such as oxidative and metabolic stress (ROS and oxidative phosphorylation, and lipid transport and metabolism), with concomitant downregulation of DNA-damage-repair and mitochondrial-response pathways (Fig. 3a). All of the Sen cell types had more DNA-damage foci compared with NSen or basal cells (γH2AX immunostaining) (Fig. 3b), and ROS-induced DNA damage and telomeric DDR (Extended Data Fig. 4k,l). 3 d.p.i. Sen cells had more intense CellRox (ROS) staining compared with NSen (or basal) counterparts (Fig. 3b), which may relate to their mitochondrial dysfunction (Fig. 3a).

To assess the causality of the high ROS levels in driving injury-induced senescence, we sorted ROS^high^ and ROS^low^ SCs and FAPs from muscles at 1 d.p.i. (before the appearance of senescent cells). ROS^high^ cells sorted at 1 d.p.i. presented higher levels of 8-oxoguanine compared with ROS^low^ cells (Fig. 3c). Moreover, ROS^high^ cells, but not ROS^low^ cells, became senescent in culture, and ROS inhibition with the antioxidant *N*-acetylcysteine (NAC) blocked senescence entry of ROS^high^ cells (Fig. 3d). During regeneration, senescence was reduced by inhibiting ROS (Fig. 3e). Sen cells showed altered lipid-transport and lipoprotein-remodelling traits (Fig. 3a), consistent with the ability of oxidative stress to generate lipotoxicity that triggers inflammation and fibrosis^30^.

To analyse the sole effect of ageing on resting-muscle cells, we compared basal cells of old and young mice and found upregulation of the immune-inflammatory response, DNA-damage and cell-cycle arrest, lipid metabolism, matrix remodelling and insulin-signalling genes, and downregulation of mitochondrial genes in the old mice (Extended Data Fig. 7b). Basal cells from old mice had more DNA-damage foci compared with their young counterparts (Fig. 1c). This coincided with higher ROS levels in cells of the old mice^6,9^. Matrix deposition (Extended Data Fig. 7c) and expression of cell-cycle inhibitors and inflammatory factors were also higher in resting muscles of old mice (Extended Data Fig. 7d), consistent with the concept of inflammageing^4^. Thus, injury and ageing led to the accumulation of stressors and activation of inflammatory/fibrotic pathways that primed old niche cells for senescence, resulting in a deeper senescent state after injury.

## Two hallmarks define senescence in vivo

**Extended Data Fig. 8:**
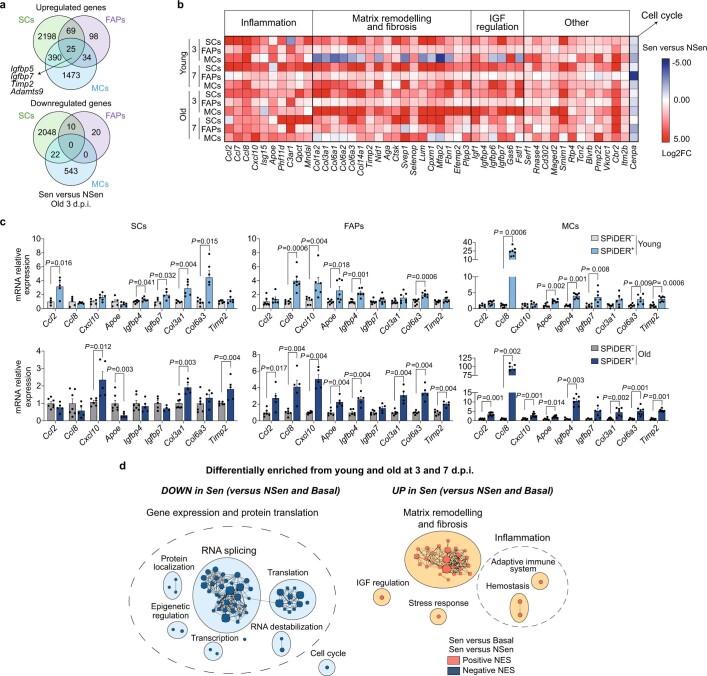
Commonly differentially expressed genes in senescence transcriptomes and qPCR validation of selected genes. **a**) Venn-diagram showing the overlap between differentially expressed genes in SCs, FAPs, and MCs from old mice at 3 d.p.i. (Sen vs NSen were compared, FDR < 0.05). **b**) Heatmap of commonly regulated genes in Sen SCs, FAPs, and MCs from young and old mice at 3 and 7 d.p.i. (FDR <0.05 in at least 8/12 comparisons). **c**) mRNA quantification by RT-qPCR of indicated genes in SPiDER^+^ and SPIDER^–^ SCs, FAPs, and MCs isolated from regenerating muscles of young (*n* = 4 mice in *Ccl2* for SCs^NSen^, 5 mice in *Ccl8*, *Cxcl10*, *Apoe*, *Igfbp7*, *Col3a1* for SCs^NSen^, *Ccl2*, *Ccl8*, *Igfbp7* for SCs^Sen^, 6 mice in *Igfbp4*, *Col6a3*, *Timp2* for SCs^NSen^, *Cxcl10*, *Apoe*, *Col3a1*, *Col6a3*, *Timp2* for SCs^Sen^, *Cxcl10* for FAPs^NSen^, *Ccl2*, *Cxcl10*, *Igfbp4* for FAPs^Sen^, *Ccl2*, *Cxcl10*, *Igfbp4*, *Igfbp7*, *Col6a3* for MCs^NSen^, *Cxcl10*, *Col3a1*, *Col6a3* for MCs^Sen^ and 7 mice for the rest of the genes and groups) and old mice (*n* = 4 mice in *Cxcl10* and *Igfbp7* for SCs^Sen^ and *Igfbp7* for MCs^NSen^, 5 mice in *Igfbp7* for SCs^NSen^, *Ccl2*, *Ccl8*, *Apoe*, *Igfbp4*, *Col3a1*, *Col6a3*, and *Timp2* for SCs^Sen^, *Igfbp4* for MCs^NSen^ and all genes for FAPs^Sen^, 7 mice for *Ccl2*, *Ccl8*, *Cxcl10*, *Apoe*, *Igfbp4*, *Col3a1*, and *Col6a3* for SCs^NSen^, *Ccl2*, *Apoe*, *Igfbp4*, *Igfbp7*, *Col3a1*, *Col6a3*, *Timp2* for MCs^Sen^, and 6 mice for the rest of the genes and groups) at 3 d.p.i. **d**) Common clusters of gene sets (GSEA) from Sen vs NSen and Sen vs basal SCs, FAPs and MCs from young and old mice at 3 and 7 d.p.i. Gene sets were considered common with FDR < 0.25 in at least 8/12 comparisons for Sen vs NSen and Sen vs basal. Node size is proportional to the number of genes identified in each gene set. Grey edges indicate gene overlap. Results are displayed as mean ± s.e.m.; *P* values were calculated by Mann–Whitney *U*-test (**c**). Source data

We next searched for commonly regulated traits in Sen cells across all ages and time points. Despite gene-expression heterogeneity (Extended Data Figs. 5e and 8a), 47 differentially expressed genes were largely conserved among conditions (Extended Data Fig. 8b), including pro-inflammatory cytokines (such as *Ccl2*, *Ccl7* and *Ccl8*), matrix/remodelling components (such as *Col1a2*, *Col3a1* and *Timp2*) and insulin growth factor (IGF) regulators (such as *Igfbp4*, *Igfbp6* and *Igfbp7*), that were previously linked to senescence. Expression of the pro-inflammatory/pro-fibrotic genes *Ccl2*, *Ccl7*, *Igf1*, *Igfbp4* and *Timp2* was also detected by scRNA-seq mapping (Fig. 1g). Key genes were validated using quantitative PCR with reverse transcription (RT–qPCR; Extended Data Fig. 8c).

**Fig. 4:**
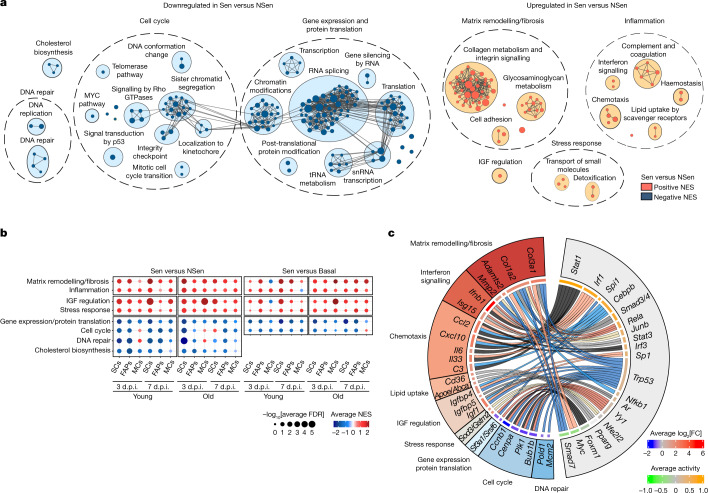
Two major common hallmarks define senescent cells across cell types, regeneration stage and lifespan. **a**, Clusters of gene sets (GSEA) differentially enriched from Sen versus NSen SCs, FAPs and MCs from young or old mice at 3 or 7 d.p.i. Gene sets were considered to be common with FDR < 0.25 in at least 8 out of 12 comparisons. Node size is proportional to the number of genes identified in each gene set. The grey edges indicate gene overlap. **b**, Common clusters of gene sets (GSEA) from Sen versus NSen and Sen versus basal SCs, FAPs and MCs from young or old mice at 3 or 7 d.p.i. Gene sets were considered to be common with FDR < 0.25 in at least 8 out of 12 comparisons for Sen versus NSen and Sen versus basal. **c**, Chord diagram showing transcription factors that regulate the differentially expressed genes in Sen versus NSen and their categories. The green-to-orange scale indicates the average predicted transcription factor activity. The blue-to-red scale indicates the average base 2 logarithm fold change (log_2_[FC]) of a transcription factor target in Sen versus NSen cells. Chord width is proportional to the significance (−log_10_[FDR]) of canonical pathway and Gene Ontology biological process (GO:BP) enrichment (gprofiler2) within a given functional category. Source data

Pathway-enrichment analysis showed upregulation of two major functions: inflammation (complement and coagulation, chemotaxis, interferon signalling and lipid uptake by scavenger receptors) and matrix remodelling/fibrosis (extracellular matrix glycoproteins and cell adhesion) (Fig. 4a). These two hallmarks are conserved in replicative senescence in vitro^31^. Minor conserved traits were related to stress responses (cell detoxification and small-molecule transport) and IGF regulation (IGF transport/uptake by IGFBPs) (Fig. 4a). By contrast, basic cell machinery processes were downregulated across conditions, with reduced gene expression, splicing, translation, mitotic cell-cycle processes, and DNA replication and repair functions (Fig. 4a). A comparison of Sen cells with their NSen and basal counterparts simultaneously showed that core senescence hallmarks were maintained (Fig. 4b and Extended Data Fig. 8d), excluding that they were due to the cell-growth arrest (quiescence) state per se.

## Chromatin analysis of senescent cells

**Extended Data Fig. 9:**
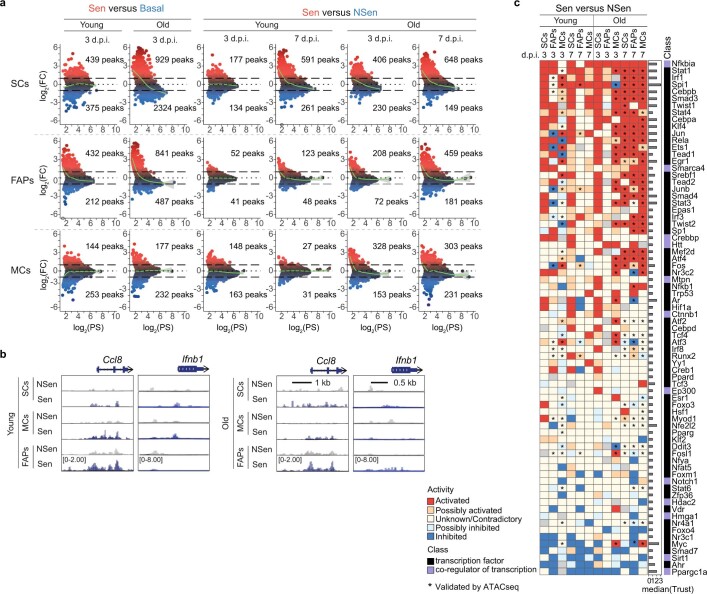
ATACseq analysis of the different senescent cell types throughout life. **a**) *Differential ATAC-seq peaks for Sen vs NSen* SCs, FAPs, and MCs from old mice at 3 and 7 d.p.i. Left: log_2_FC plotted against log_2_-transformed average peak intensity (peak score); the green line shows the position of each average peak score on the log_2_FC axis; the number of peaks with log_2_FC >1 or ≤1 is indicated. **b**) Normalized ATAC-seq signal profiles in the indicated gene regions from Sen vs NSen SCs, FAPs, and MCs from young and old mice at 7 d.p.i. **c**) Heatmap representing transcription factors and co-regulators of transcription enriched in at least 11/12 comparisons for Sen vs NSen with average Trust score > 1 (see Methods). Colour codes reflect the activity predicted based on analysis of differential expression (DESeq2), upstream regulators analysis (QIAGEN’s IPA) and motif enrichment analysis in RNA-seq and ATAC-seq data. Source data

Transcription factor enrichment analysis in Sen cells in all conditions revealed enrichment occupancy of key regulators of inflammation and the SASP, including NF-κB^32^, C/EBPβ^33^ and STAT1/3 (ref. ^34^), and of matrix remodelling/fibrosis, including SMAD3/4 (and inhibition of SMAD7)^35^ (Fig. 4c). We also performed transposase-accessible chromatin high-throughput sequencing (ATAC-seq) analysis of Sen and NSen FAPs, MCs and SCs at early and late regeneration phases (and in basal cells) from young and old mice, and analysed the promoter accessibility of the genes that define senescence in our transcriptomic data. By comparing the signal for each ATAC-seq peak, we observed total reduced chromatin accessibility in Sen cells (Extended Data Fig. 9a). In Sen cells of old mice, promoters with medium peaks were more accessible. As the low number of cells used for ATAC-seq limited its resolution, we restricted our analysis to evaluate the overall chromatin accessibility between Sen and NSen cells. We detected an increased peak signal at proximal promoters of the *Ifnb1* and *Ccl8* genes in Sen cells, correlating with increased expression (Extended Data Fig. 9b). Motif-enrichment analysis of ATAC-seq data validated the transcription factors predicted by RNA-seq (NF-κB, C/EBPβ, STAT1/3, SMAD3/4) (Extended Data Fig. 9c), reinforcing that senescent cells within the muscle niche were of an inflammatory/fibrotic nature, regardless of age or time after injury.

## A senescent aged-like inflamed niche

**Fig. 5:**
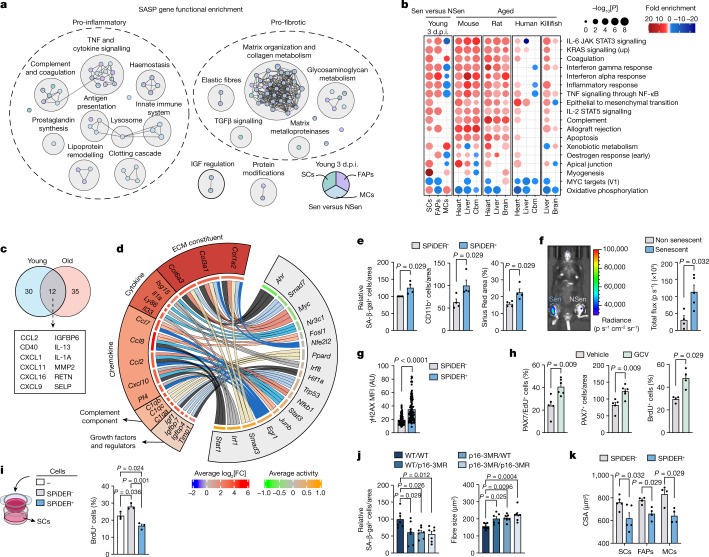
Senescent cells create an aged-like microenvironment in young regenerative niches through pro-inflammatory and pro-fibrotic factor secretion. **a**, SASP-related gene set enriched clusters from Sen SCs, FAPs and MCs of young mice at 3 d.p.i. (FDR < 0.05). The grey edges indicate gene overlap. Differentially upregulated genes (FDR < 0.05) were considered to be SASP genes when overexpressed in Sen versus their NSen populations. **b**, Comparison of enrichments for differential RNA expression in tissues from aged mice, rats, humans, killifish and senescent populations from young 3 d.p.i. muscles. Cbm, cerebellum. **c**, The overlap between secreted proteins in cells of young and aged muscle. **d**, Chord diagram showing transcription factors that regulate SASP genes and their categories in Sen versus NSen. Chord width is proportional to the significance (−log_10_[FDR]) of GO molecular function (GO:MF) cluster enrichment. ECM, extracellular matrix. **e**, SPiDER^+^ and SPiDER^−^ cells from 3 d.p.i. young muscle were stained with Dil and transplanted into the preinjured TA of recipient mice for 4 days. *n* = 4 mice per group. Quantification of SA-β-gal^+^ and CD11b^+^ cells, and Sirius Red staining. **f**, As described in **e**, but senescent and non-senescent C2C12 cells were transplanted into young p16-3MR mice for 5 days. Images and quantification of luciferase-activity. *n* = 4 mice (non-senescent) and *n* = 5 mice (senescent). **g**, As described in **e**, but quantification of γH2AX in Pax7^+^ cells (*n* = 109 cells (SPiDER^−^) and *n* = 100 cells (SPiDER^+^)). **h**, CTX-injured p16-3MR mice were treated daily with GCV or vehicle until 4 d.p.i. (*n* = 5 TA muscles from 3 mice (vehicle) and *n* = 6 TA muscles from 3 mice (GCV)). Left, images and quantification of EdU or Pax7 staining. Right, BrdU incorporation in SCs in vitro. At 3 d.p.i., SCs were sorted and cultured for 3 days. *n* = 4 mice per group. **i**, SPiDER^−^ SCs were isolated from 3 d.p.i. muscles, and cultured for 3 days in Transwells with total SPiDER^+^ or SPiDER^−^ cells or culture medium, and analysed for BrdU incorporation. *n* = 3 mice per group. **j**, EDL muscles from WT or p16-3MR-donor mice were transplanted into WT or p16-3MR recipient mice (or vice versa). The recipient mice were treated daily with GCV, and regeneration was analysed at 7 d.p.i. The CSA of MYH3^+^ fibres (*n* = 8 (WT/WT), *n* = 6 (p16-3MR/WT) and *n* = 7 (other groups) mice) and SA-β-gal^+^ cells (*n* = 7 mice per group). **k**, SPiDER^+^ and SPiDER^−^ SCs, FAPs or MCs (*n* = 5 mice (SCs) and *n* = 4 mice (FAPs and MCs)) transplanted as in **e**. The CSA of MYH3^+^ fibres. For **e**–**k**, data are mean ± s.e.m. *P* values were calculated using Tukey’s tests (**i** and **j**) and Mann–Whitney *U*-tests (**e**–**h** and **k**). Source data

**Extended Data Fig. 10:**
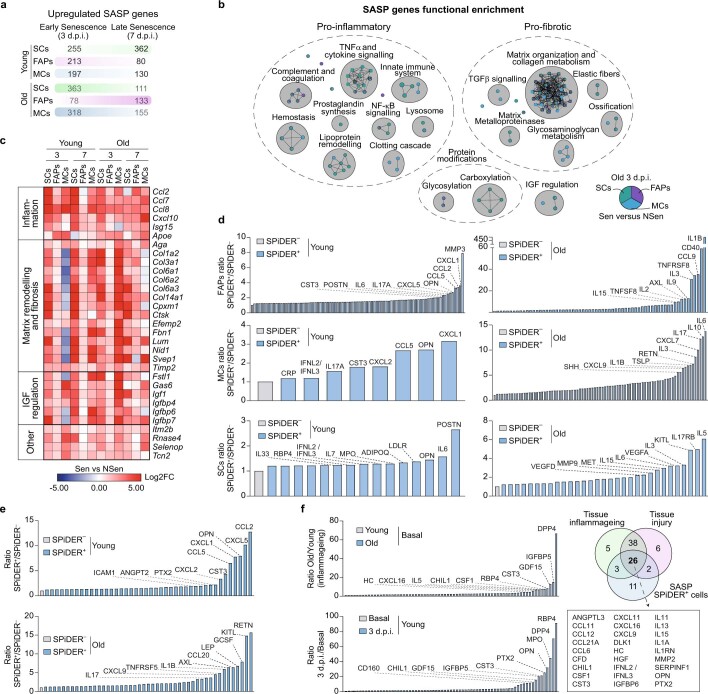
SASP transcriptome and SASP cytokine array analyses of the different senescent cell types throughout life. **a**) Scheme indicating the number of upregulated SASP genes in SCs, FAPs, and MCs from young and old mice at 3 and 7 d.p.i. (FDR <0.05). **b**) Clusters of gene sets enriched in SASP-related genes from Sen SCs, FAPs, and MCs from old mice at 3 d.p.i. (g:Profiler web server, FDR <0.05). Node size is proportional to the number of genes identified in each gene set. Grey edges indicate gene overlap. SASP genes were identified using different published databases (see methods). Differentially upregulated genes (FDR < 0.05) were considered as “SASP genes” when overexpressed in Sen populations vs their NSen counterparts. **c**) Heatmap of upregulated SASP genes in Sen SCs, FAPs and MCs from young and old mice at 3 and 7 d.p.i. (FDR <0.05 in at least 8/12 comparisons). **d**) SPiDER^+^ and SPiDER^–^ SCs, FAPs and MCs, freshly sorted from regenerating muscle tissue at 3 d.p.i., were cultured for 24 h in serum-deprived media, conditioned media collected, and protein levels assessed by cytokine array (*n* = pool of 4 mice/group). Graphs show the top 10 proteins whose levels were increased in SPiDER^+^ cells (compared to the SPiDER^–^). **e**) Cytokine array of freshly sorted SPiDER^+^ or SPiDER^–^ cells from regenerating muscle at 3 d.p.i. from young or old mice cultured for 24 hours in serum-deprived media, then the conditioned media were collected and the levels of the indicated protein were assessed. Graphs represent the top 10 proteins whose levels were increased in the comparison (*n* = pool of 4 mice/group). **f**) (left) Cytokine array analysis of whole muscle secretome from (top) resting (basal) muscles comparing old and young mice, and (bottom) injured compared to basal muscles from young mice (*n* = pool of 2 mice/group). Graphs represent the top 10 proteins whose levels were increased in the comparison. (right) Venn-diagram showing the overlap between secreted proteins during ageing, injury-induced regeneration and secreted proteins by isolated young SPiDER^+^ cells in **e**. Common secreted proteins are indicated. Source data

To understand how senescent cells impair muscle regeneration, we studied their SASP after injury and during ageing. We selected differentially expressed genes with extracellular or secreted protein products in Sen versus NSen cells. Depending on cell type and conditions, the number of SASP components ranged from 78 to 363, highlighting SASP diversity (Extended Data Fig. 10a). Pathway enrichment identified two major functions: (1) inflammation, including complement and coagulation, innate-immune system, lipoprotein remodelling and cytokine and TNF/NF-κB signalling (*Ccl2*, *Ccl7*, *Ccl8* and *Isg15*); and (2) fibrosis, including matrix organization and collagen metabolism, and TGFβ signalling (*Col3a1*, *Col6a2* and *Timp2*) (Fig. 5a and Extended Data Fig. 10b,c). Some of these genes were commonly identified in scRNA-seq (Fig. 1g). IGF regulation by IGF-binding proteins (*Igfbp4*, *Igfbp6*, *Igfbp7* and *Igf1*) was also present (Fig. 5a and Extended Data Fig. 10b,c). Thus, the major SASP features corresponded with the universal hallmarks of senescent cells in vivo (Fig. 4a).

We next compared the transcriptomes of ageing tissues from various species (including humans) with those of Sen cells from injured young muscles, and found an increase in inflammatory pathways (interferon, complement and cytokine and TNF/NF-κB signalling) (Fig. 5b), consistent with previously published datasets^36–38^. A secreted-protein array-based assay confirmed the secretion of inflammatory/matrix-remodelling proteins in sorted Sen cells (from individual cell types or in combination) from young muscle (Fig. 5c and Extended Data Fig. 10d,e). Many of these proteins were also secreted by Sen cells from injured aged muscle (CCL2, IGFBP6, CD40, IL13, CXCL1, IL1A, CXCL11 and MMP2) (Fig. 5c and Extended Data Fig. 10d,e). In whole-tissue analysis, many secreted SASP proteins were commonly upregulated in injured young muscles and in non-injured aged muscles compared with non-injured young muscles (Extended Data Fig. 10f), indicating a shared inflammatory secretome in injured young tissue and in basal ageing conditions. Thus, the SASP of Sen cells, transiently present in injured young muscles, mimics aged-like inflammageing, which is exacerbated in injured aged muscle.

**Extended Data Fig. 11:**
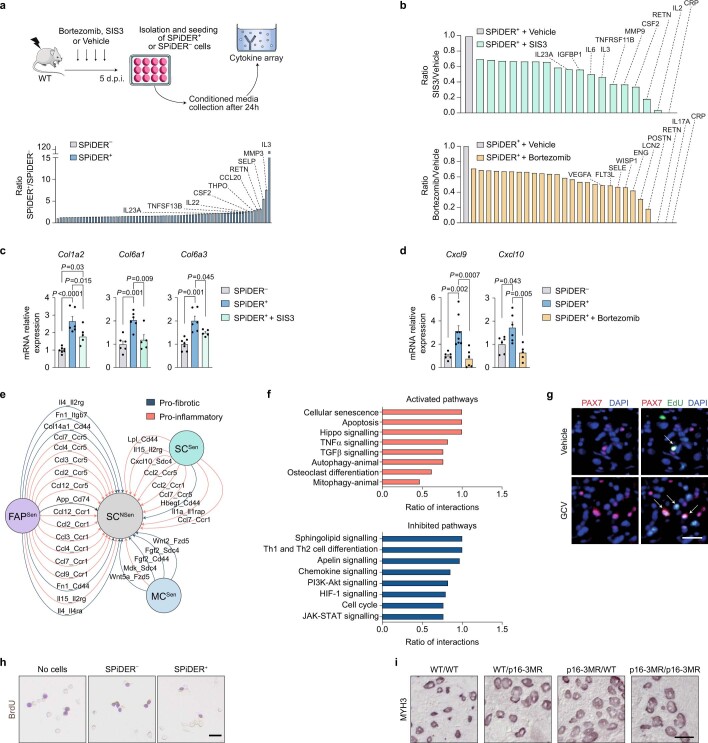
Role of NF-κB and Smad3 in the inflammatory SASP in regenerating muscles. Analysis of SASP and its effect on muscle stem-cell expansion. **a**) Mice were subjected to CTX injury and treated with either vehicle, bortezomib or SIS3 during the course of regeneration and analysed at 5 d.p.i.. Strategy schematic and cytokine array of freshly sorted SPiDER^+^ or SPiDER^–^ cells from regenerating muscle at 5 d.p.i. are shown (*n* = pool of 4 mice/group). Graphs represent the top 10 proteins whose levels were increased in the comparison. **b**) Cytokine array of freshly sorted SPiDER^+^ or SPiDER^–^ cells from regenerating muscle at 5 d.p.i. from mice treated with SIS3, bortezomib or vehicle (*n* = pool of 4 mice/group for vehicle and SIS3 and pool of 3 mice/group for bortezomib). Graphs represent the top 10 proteins whose levels were decreased in the comparison. **c**) Expression levels of the indicated genes analysed by RT-qPCR in vehicle- and SIS3-treated mice (*n* = 5 mice in *Col6a1* and *Col6a3* for SPiDER^+^+SIS3, 7 mice in *Col1a2* and *Col6a3* for SPiDER^−^, and 6 mice for the rest of the genes and groups). **d**) Expression levels of the indicated genes analysed by RT-qPCR in vehicle and bortezomib-treated mice (*n* = 5 mice in *Cxcl10* for SPiDER^+^+bortezomib, 8 mice in *Cxcl9* for SPiDER^+^, and 6 mice for the rest of the genes and groups). **e**) Cytoscape network showing ligand-receptor (L-R) interactions between Sen populations and NSen SCs from old mice at 3 d.p.i. predicted by a modified version of FunRes. **f**) Major activated and inhibited KEGG pathways predicted by SPIA in NSen SCs downstream the predicted interactions showed in **e**. Ratio of interactions represents the proportion of L-R that induce the pathway of interest. **g**) p16-3MR mice were injured with CTX and daily treated with vehicle or GCV from the day of injury to 4 d.p.i. Representative images of EdU and Pax7 staining, arrows indicate double-positive cells (related to Fig. 5h). **h**) SPiDER^–^ SCs were isolated at 3 d.p.i. from regenerating muscles of young mice, then cultured for 3 days in transwells with total SPiDER^+^, SPiDER^–^ cells, or culture medium. After 3 days of culture, SC proliferation was assessed by BrdU incorporation. Representative images of BrdU staining are shown (related to Fig. 5i). **i**) EDL from either WT or p16-3MR donor mice were transplanted into WT or p16-3MR recipient mice or vice versa. Recipient mice were treated every day with GCV, and muscle regeneration was analysed at 7 d.p.i. Representative images of MYH3 staining are shown (related to Fig. 5j). Scale bars: 20 μm (**g** and **i**); 10 μm (**h**). Results are displayed as mean ± s.e.m.; *P* values were calculated by Tukey’s test (**c** and **d**). Source data

Functional profiling of SASP transcription factors confirmed the association of NF-κB, STAT1/3 and SMAD3/4 with the identified inflammatory and matrix-related SASP genes (Fig. 5d). To assess the role of NF-κB and SMAD3 in the inflammatory SASP in regenerating muscles, we treated young mice with either NF-κB (bortezomib) or SMAD3 (SIS3) inhibitors after injury. Both bortezomib and SIS3 diminished the expression of pro-inflammatory and pro-fibrotic SASPs in sorted SPiDER^+^ cells, on the basis of analyses using a secreted-protein array and gene-transcription assays (Extended Data Fig. 11a–d), suggesting that NF-κB and SMAD3 link injury-induced senescence and inflammageing in vivo.

## SASP reduces muscle stem cell expansion

To assess how the SASP affects muscle regeneration (and especially muscle SCs), we reconstructed ligand–receptor interactions between Sen cells (producing SASP ligands) and NSen SCs (with cognate receptors) using a modified FunRes algorithm^39^; this revealed predominantly inflammatory interactions (Extended Data Fig. 11e). A signalling pathway impact analysis (SPIA)^40^ of transcription factors involved in these interactions revealed that the SASP produced by all Sen cells activated downstream signalling pathways in receiving NSen SCs (senescence, apoptosis and inflammatory responses) and inhibited cell-cycle and proliferative pathways (MAPK and AKT signalling) (Extended Data Fig. 11f). Thus, SASP components might provoke either proliferative arrest or paracrine senescence in NSen SCs. To test these predictions, we transplanted sorted Sen (SPiDER^+^) and NSen (SPiDER^−^) fractions, labelled with Dil, into preinjured muscles of recipient mice. In contrast to transplanted Dil-labelled NSen cells, transplanted Dil-labelled Sen cells increased the number of senescent cells in the host tissue, induced inflammatory-cell recruitment and fibrosis, and delayed regeneration (Figs. 2d and 5e). This paracrine senescence induction was confirmed by transplantation of ex vivo induced senescent (versus non-senescent) WT cells into preinjured muscles of p16-3MR mice (Fig. 5f). Notably, transplantation of sorted SPiDER^+^ and SPiDER^−^ into non-injured muscles was sufficient to induce DNA damage in endogenous SCs (Fig. 5g). By contrast, GCV-treated p16-3MR mice showed higher numbers of proliferating and total SCs within the regenerative-muscle niche (Fig. 5h; Extended Data Fig. 11g). Consistently, SCs sorted from GCV-treated p16-3MR mice had a higher proliferation ability ex vivo compared with SCs from vehicle-treated mice (Fig. 5h) and, in Transwell assays, Sen cells reduced the proliferation of NSen SCs (Fig. 5i and Extended Data Fig. 11h). Thus, senescent cells restrained muscle regeneration through paracrine pro-inflammatory and pro-fibrotic SASP functions that blunted SC proliferation.

To examine the relative contribution to regeneration of senescent cells derived either from tissue-resident cells or from the blood, we used a whole-muscle grafting model^5^, in which the extensor digitorum longus (EDL) muscle from one mouse was grafted onto the tibialis anterior (TA) muscle of a recipient mouse. In this model, the transplanted EDL undergoes de novo myogenesis at the expense of its own SCs, while recruited bone-marrow-derived cells come from the host. EDL grafting combined with daily GCV-mediated senescent-cell depletion revealed that p16-3MR EDL grafts in WT host mice, or WT EDL grafts in p16-3MR hosts, had larger regenerating myofibres compared with WT EDL grafts in WT mice (Fig. 5j and Extended Data Fig. 11i), confirming that senescent cells from EDL-resident SCs and FAPs, and from blood cells, hampered muscle regeneration. Similar detrimental effects were seen after transplantation of equal numbers of SPiDER^+^ SCs, FAPs or MCs, separately or combined (Figs. 2d and 5k), supporting that the SASP was not beneficial for regeneration even if acting transiently.

## CD36 regulates SASP production

**Fig. 6:**
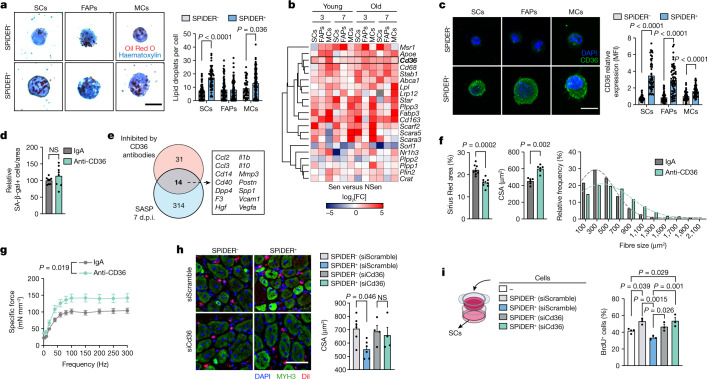
CD36 neutralization improves muscle regeneration through a senomorphic action. **a**, SPiDER^+^ and SPiDER^−^ populations from 3 d.p.i. muscle stained with Oil Red O and haematoxylin. Images and lipid-droplet quantification. *n* = 90 SCs (NSen), 51 SCs (Sen), 89 FAPs (NSen), 90 FAPs (Sen), 45 MCs (NSen) and 94 MCs (Sen). **b**, Heat map showing lipid-transport-related genes that are differentially expressed in at least 3 out of 12 comparisons between Sen and NSen cells. The colour indicates the log_2_-transformed fold change in expression. **c**, Images and CD36 quantification in SPiDER^+^ and SPiDER^−^ populations from 3 d.p.i. muscles. *n* = 44 SCs (NSen), 50 SCs (Sen), 56 FAPs (NSen), 62 FAPs (Sen), 55 MCs (NSen) and 56 MCs (Sen). **d**, SA-β-gal^+^ cells in the injured TA area of aged mice (treated with anti-CD36 antibodies or control IgA). *n* = 8 TA from 4 mice for both groups. **e**, The overlap between SASP-upregulated genes in 7 d.p.i. senescent populations of old mice, and those reduced by anti-CD36 treatment. **f**, The CSA and frequency distribution of MYH3^+^ fibre size (*n* = 6 TA from 4 mice for both groups) and Sirius Red staining (*n* = 8 TA from 4 mice for both groups) of 7 d.p.i. TA from old mice treated with anti-CD36-antibody antibodies or control IgA. **g**, Injured EDL of old mice treated with anti-CD36 antibodies or control IgA. (*n* = 6 EDL from 3 mice (IgA) and *n* = 5 EDL from 4 mice (anti-CD36)). Force–frequency curves are shown. **h**, SPiDER^+^ and SPiDER^−^ cells from 3 d.p.i. muscles were transfected with siCd36 or siScramble, stained with Dil and transplanted into the preinjured recipient TA. Images and the CSA of MYH3^+^ fibres 4 days after transplantation. *n* = 6 (siScramble-treated SPiDER^+^ and SPiDER^−^ cells) and *n* = 4 (siCd36-treated SPiDER^+^ and SPiDER^−^ cells) mice. **i**, SCs (from 3 d.p.i. muscles) were cultured for 3 days in Transwells with senescent or non-senescent C2C12 cells that were previously treated with siCd36 or siScramble, or without cells. BrdU incorporation is shown. *n* = 4 (empty Transwell) and *n* = 3 (other groups) mice. Scale bars, 10 μm (**a** and **c**) and 50 μm (**h**). For **a**, **c**, **d** and **f**–**i**, data are mean ± s.e.m. *P* values were calculated using Mann–Whitney *U*-tests (**a**, **c** and **f**), mixed-effects analysis (**g**) and Tukey’s tests (**h** and **i**); NS, not significant. Source data

**Extended Data Fig. 12:**
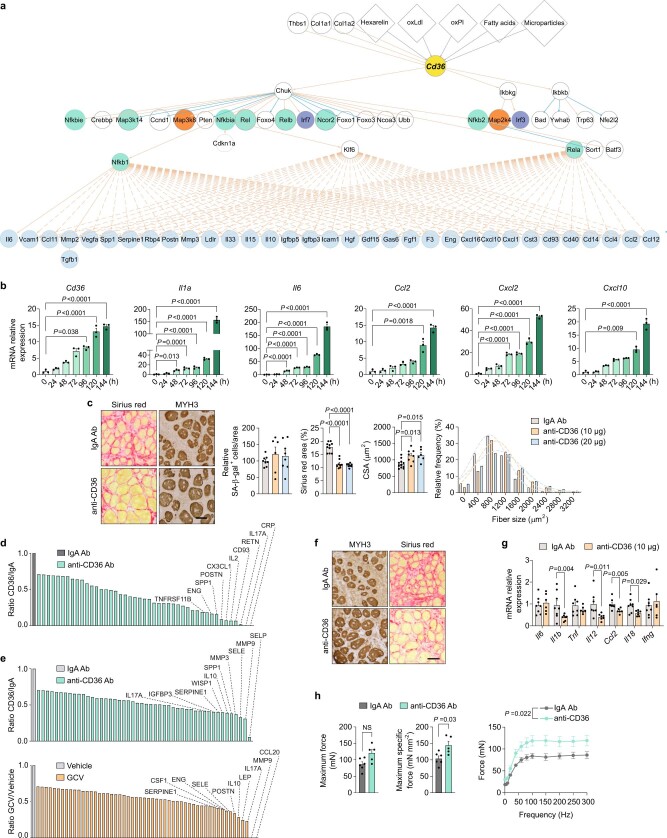
Analysis of the CD36 role in SASP production. Effects of CD36 inhibition. **a**) Subnetwork of significant *Cd36* upstream and downstream signalling interactions pulled out from FunRes global signalling interaction network for Sen SCs population at 3 d.p.i. Green nodes are related to NF-κB cascade, orange to MAPK signalling and violet to interferon regulatory factors (IRFs). **b**) C2C12 cells were treated with etoposide to induce cellular senescence and harvested at the indicated time points. Graphs show relative mRNA expression levels of *Cd36* and SASP-related genes normalized to untreated C2C12 cells at different times after etoposide treatment (*n* = 3 experiments). **c**) TA muscles of young mice were subjected to CTX injury and mice were treated with control IgA or anti-CD36 antibody from 3 to 7 d.p.i. once per day. Representative images of MYH3 and Sirius Red staining and quantification of SA-β-gal^+^ cells (*n* = 8 TA from 7 mice for IgA, 6 TA from 4 mice for anti-CD36^10μg^, and 8 TA from 4 mice for anti-CD36^20μg^), mean CSA (*n* = 11 TA from 7 mice for IgA, 8 TA from 4 mice for anti-CD36^10μg^, and 6 TA from 4 mice for anti-CD36^20μg^) and frequency distribution analysis of MYH3^+^ fibres (*n* = 7 mice for IgA, 4 mice for anti-CD36^10μg^, and 4 mice for anti-CD36^20μg^) and Sirius Red staining (*n* = 10 TA from 6 mice for IgA, 8 TA from 4 mice for anti-CD36^10μg^ and anti-CD36^20μg^) in muscle cryosections. **d**) Freshly sorted SPiDER^+^ cells from IgA or anti-CD36 antibody-treated old mice at 7 d.p.i. were cultured for 24 h in serum-deprived media, conditioned media was collected and protein levels were assessed by cytokine array. Quantification showing the proteins whose levels were reduced by 30% in the presence of anti-CD36 antibody (*n* = pool of 4 mice/group). **e**) Cytokine array analysis of whole muscle secretome from p16-3MR mice treated with (top) IgA or anti-CD36 antibody or (bottom) GCV or vehicle, as indicated before (*n* = pool of 3 mice for anti-CD36 and pool of 2 mice for the rest of the groups). Graphs represent the top 10 proteins whose levels were decreased in the comparison. **f**) Representative pictures of MYH3 and Sirius Red staining of regenerating TA muscles from IgA or anti-CD36 antibody-treated old mice at 7 d.p.i. (related to Fig. 6f). **g**) As in **c**, mRNA expression levels of the indicated genes by RT-qPCR (*n* = 6 TA from 3 mice in *Il6* and *Ifng* for anti-CD36, 6 TA from 4 mice in *Tnf* and *Ccl2* for anti-CD36, 7 TA from 4 mice in *Il1b*, *Il12*, *Il18* for anti-CD36 and *Ccl2* and *Il12* for IgA and 8 TA from 4 mice for the rest of the genes and groups). **h**) EDL muscles of old mice were injured with CTX and mice were treated with IgA or anti-CD36 antibodies from 3 to 10 d.p.i. Maximum and specific force measurements and force-frequency curve are shown (*n* = 6 EDL from 3 mice for IgA Ab-treated and 5 EDL from 4 mice for anti-CD36-treated). Scale bars 50 μm. Results are displayed as mean ± s.e.m.; *P* values were calculated by Dunnet’s test (**b**), Tukey’s test (**c**), two-way ANOVA (**h**, force-frequency curve) and Mann–Whitney *U*-test (**g** and **h**). Source data

Given the heterogeneity of SASPs in vivo, we searched for a broad SASP-targeting approach. Lipid transport, which is tightly associated with inflammatory responses^41^, was consistently included in the inflammatory hallmark of Sen cells (Fig. 4a) and the SASP (Fig. 5a and Extended Data Fig. 10b) in all conditions. Sen cells had more lipid droplets compared with NSen cells (Fig. 6a). Numerous lipid metabolism and lipid-transport genes, including *Fabp3*, *Apoe*, *Star*, *Lpl*, *Cd68* and *Cd36*, were upregulated in all Sen cells (Fig. 6b). As lipid uptake and CD36 are related to SASP in vitro^42,43^, CD36 might also be related to the SASP in vivo. FunRes generated a CD36 signalling network that predicted downstream activation of NF-κB and other inflammation/stress-related pathways, and downstream SASP components, such as *Il6*, *Tgfb1*, *Mmp3*, *Igfbp5*, *Ccl2* and *Cxcl10* (Extended Data Fig. 12a), suggesting that CD36 might regulate the in vivo senescence secretory program, affecting regeneration.

All three Sen cell types had higher CD36 protein expression in injured muscles (Fig. 6c). *Cd36* expression was also upregulated in etoposide-induced senescent cells in vitro (Extended Data Fig. 12b). We next analysed injured muscles from young or old mice, treated for 4 days (starting at 3 d.p.i.) with an anti-CD36 neutralizing antibody (at two distinct doses) or a control IgA antibody. CD36 blockade did not affect the number of senescent cells (Fig. 6d and Extended Data Fig. 12c), but reduced several SASP proteins (Extended Data Fig. 12d), and many coincided with SASP genes in Sen SCs, FAPs and MCs encoding chemokines, cytokines and matrix metalloproteinases (*Ccl2*, *Ccl3*, *Il1b, Il10* and *Mmp3*) (Fig. 6e). Moreover, the whole injured-muscle secretome showed lower SASP protein levels in both GCV-treated and anti-CD36-antibody-treated p16-3MR mice (Extended Data Fig. 12e). Several Sen-cell-secreted inflammatory SASPs (*Ccl2*, *Ccl4* and *Cxcl10*) that induced downstream signalling in NSen SCs in the ligand–receptor interactive network were predicted to negatively affect SC functions and regeneration (Extended Data Fig. 11e,f), and some appeared as CD36-regulated SASPs (Extended Data Fig. 12a). CD36 blockade improved regeneration in both young and old muscles (Fig. 6f and Extended Data Fig. 12c,f) while reducing inflammation (Extended Data Fig. 12g) and fibrosis (Fig. 6f and Extended Data Fig. 12c,f), and these muscles showed increased force (Fig. 6g and Extended Data Fig. 12h).

We next silenced *Cd36* in sorted Sen cells using *Cd36*-specific short interfering RNA (siCd36), with scrambled siRNA (siScramble) used as a control. After transplantation into injured muscle, siScramble-treated Sen cells delayed regeneration, whereas *Cd36*-silenced Sen cells had no negative effects (Fig. 6h). Consistent with this result, the SASPs produced by sorted Sen cells reduced SC proliferation in co-culture Transwell assays (Fig. 5i), but this effect was not observed when *Cd36* was silenced in Sen cells before co-culturing with SCs (Fig. 6i); thus, CD36 was crucial for the paracrine effects of Sen cells on muscle regeneration by regulating SASP production.

## Discussion

Proper reconstruction of injured tissue requires timely interaction among diverse cell types within the regenerative niche. Senescent cells affect tissue repair processes, but the mechanisms are largely unclear. One reason has been the technical limitations imposed by both the scarcity and the heterogeneity of senescent cells, and a lack of ‘universal’ senescence markers^3^. By establishing a sorting protocol that enriches senescent cells of distinct types (confirmed in vivo using multi-marker microscopy), we have now identified that senescent cells are integral components of the skeletal muscle niche and have demonstrated that they repress regeneration in response to distinct types of injuries in young mice, and even more strongly in aged mice.

Here we showed that senescent cells were practically absent (or in very low numbers) in unperturbed muscle tissues, even in old age, but emerged after injury. In injured muscle, senescent cells modified their normal niche counterparts by creating an aged-like inflamed microenvironment that hijacked their proliferative programs and blunted regeneration. Reducing the number of senescent cells (and therefore their inflammatory secretome) resumed stem-cell proliferation and enhanced muscle regeneration. Thus, efficient tissue regeneration involves not only constructive cellular crosstalks but also unexpectedly repressive interactions.

An important question is how senescent cells arise after tissue damage. We provide evidence that, after injury, a subset of niche cells accumulates ROS and DNA damage beyond a threshold, which leads to senescence and regenerative failure. Thus, proliferating cells in injured tissues actively repress their senescence program, thereby preserving cell integrity and fitness. This is particularly critical in old age as long-lasting cells accumulate damage (including telomere damage) over a lifetime and are therefore primed for senescence. Furthermore, the functionally weakened immune system in old age^44^ does not clear senescent cells, which may account for their persistence in damaged aged muscle; this exacerbates tissue regenerative impairment.

Senescence has been molecularly characterized mainly in vitro^10,45,46^. Through FACS-based enrichment of SA-β-gal-positive cells from injured muscle, followed by scRNA-seq, we generated a map of senescent cells in vivo, revealing three major types of senescent cells in regenerating muscle (MCs, FAPs and SCs), as well as other smaller populations. The successful enrichment of bona fide senescent cells in our method relied on restricting the FACS-isolation protocol exclusively to the highest SPiDER-β-gal expressing cells. Yet, some classical markers of senescence in vitro (such as CDKN2A, p21^CIP1^, IL-6 and IL-1) were observed in some, but not in all, senescent cells in vivo. A possible explanation is that the senescent state can be influenced in vivo by its trigger, the cell that senesces, its environment and its temporal resolution by the immune system, resulting in a wide spectrum of phenotypic features. We confirmed that MCs, FAPs and SCs (in this order of abundance) are the principal senescent cell types in regenerating muscle. Despite the variability in gene expression within senescent cells, we revealed that two major conserved universal hallmarks define senescent cells: inflammation and fibrosis. We also determined that, depending on their lineage of origin, senescent cells have unique features that are preserved throughout life, indicating that they share universal programs while maintaining identity traits, and adapt to the idiosyncrasies of their origin and age. Thus, our study provided a better definition of senescence in vivo: despite being a state of irreversible arrest, senescence encompasses features of molecular diversity and dynamism (sensitive to the pass of time and mode of injury) as well as conserved hallmarks.

Our pathway analyses revealed that inflammation is also broadly upregulated in senescent cells, which is consistent with the concept of inflammageing^4^. We detected upregulation of complement, interferon and TNF/NF-κB pathways in senescent cells, which are robustly activated with ageing across vertebrate tissues, including humans^36–38^. Notably, interferon signalling has been linked to viral infection responses and mitochondrial stress^47,48^, and to the reactivation of transposable elements, which is also linked to senescence^49^. Transcriptomic and chromatin-accessibility analyses in senescent cells in vivo revealed an association between the transcription factors NF-κB, SMAD, IRF1/3 and C/EBPβ, and the induction of inflammatory and matrix components and interferon-response genes, accounting for the deleterious SASP effects on niche cells. In this context, NF-κB and SMAD3 (signalling participants in inflammageing^4,38^) appeared to be causally involved in the pro-inflammatory/pro-fibrotic SASP production by senescent cells in young injured muscles.

Thus, our studies demonstrate that senescent cells are a decisive factor in tissue regeneration throughout life. The transformation of subsets of SCs and niche cells into permanently arrested senescent cells not only reduces the progeny available for regeneration but also, through the pro-inflammatory SASP, confers young tissue with an aged-like inflamed niche, therefore mirroring the negative effects of inflammageing. Mechanistically, the SASP induces senescence in surrounding healthy cells, which further spreads senescence and lowers stem-cell proliferation. Consistent with this, a reduction in the number of senescent cells improved regeneration as a result of attenuating inflammatory and fibrotic SASPs. Finally, we identified CD36 (a scavenger receptor that is related to lipid metabolism and inflammatory function) as a cell receptor acting as a senomorphic in vivo. Together, these results challenge the prevailing idea that cellular senescence is always beneficial when it occurs transiently in young tissues, and deleterious only when it occurs chronically during ageing or in diseased states. Notably, outside the tissue regeneration context (this study), both positive and negative roles for senescent cells have been proposed in muscle, such as in exercised muscle, during Yamanaka-factor-induced muscle reprogramming and after irradiation^17,22,50^, therefore exemplifying the complexity of the functions of senescent cells in distinct in vivo contexts.

Through the technical advancement in senescent-cell separation, our findings provide a transcriptome atlas of specific senescent-cell types in vivo (and a common SASP signature), and a conceptual explanation for the emergence, causes, definition, dynamics and consequences of senescence in vivo. We recognize limitations in our study associated to: (1) the use of distinct senescent-cell ablation methods (genetic and pharmacologic) and (2) the presence of a small percentage of non-senescent cells in the SPiDER^+^-sorted fraction. However, our findings provide evidence that senescence is a more complex array of states than previously anticipated. As senescent cells also emerge in damaged human muscle, these findings have implications for regenerative medicine, including in sarcopenia.

## Methods

### Animal models

C57Bl/6 (WT), p16-3MR (donated by J. Campisi)^18^, dystrophic mdx (DBA/2-background) and mdx/p16-3MR (dystrophic mdx mice crossed with p16-3MR mice) were bred and aged at the animal facility of the Barcelona Biomedical Research Park (PRBB), housed in standard cages under 12 h–12 h light–dark cycles and fed ad libitum with a standard chow diet. All of the experiments followed the principle of the ‘three Rs’—replacement, reduction and refinement according to Directive 63/2010 and its implementation in the Member States. All of the procedures had authorization from the PRBB Animal Research Ethics Committee (PRBB-CEEA) and the local government (Generalitat de Catalunya) and were conducted according to the European Directive 2010/63/EU and Spanish regulations RD 53/2013. Both male and female mice were used in each experiment unless stated otherwise. Live colonies were maintained and genotyped according to the Jackson Laboratories’ guidelines and protocols. The mice were housed together, their health was monitored daily for sickness symptoms (not age-related weight loss and so on) and they were euthanized immediately at the clinical end point when recommended by veterinary and biological service staff members. Mice were randomly allocated to experimental or treatment groups. No blinding was used. No statistical methods were used to predetermine the sample size. For PCR genotyping, the following primers were used: p16-3MR-1, 5′-AACGCAAACGCATGATCACTG-3′; and p16-3MR-2, 5′-TCAGGGATGATGCATCTAGC-3′. Positive mice show a band at 202 bp.

### Human biopsies

Human muscle biopsy samples from the vastus lateralis muscle of patients undergoing surgery were obtained from the biobank of the EU/FP7 Myoage Consortium, as previously reported^5,6^. Ethical approval was received from the local ethics committees at each of the five research centres of the Consortium. All of the participants provided written informed consent and were medically screened before participation. The biobanked muscle tissue had been directly frozen in melting isopentane and stored at −80 °C until analysed. Damaged areas were identified by morphological criteria on the basis of the presence of infiltrating mononuclear cells. Data are from female patients aged 69, 82, 80, 89 or 85 years old; the average age was 81 ± 7.5 years.

### In vivo treatments

Quercetin (USP, 1592409; 50 mg kg^−^^1^) and dasatinib (LC Laboratories, D-3307; 5 mg kg^−^^1^) were administered orally (by gavage). Control mice were administered with an equal volume of vehicle (10% ethanol, 30% polyethilenglicol and 60% phosal). GCV (Sigma-Aldrich, G2536-100MG; 25 mg kg^−^^1^) was injected intraperitoneally (i.p.). Anti-CD36 antibodies (Cayman Chemical, 10009893; 10 µg or 20 µg in young mice and 20 µg in old mice) diluted in PBS was administered via i.p., and control mice received an equal dose of IgA control antibodies (Southern Biotech/Bionova, 0106-14). Treatments with GCV, senolytics and CD36 were administered daily for 4–7 consecutive days as indicated in the figure legends. NAC (Sigma-Aldrich, A9165; 0.01 g ml^−1^) was added into drinking water (exchanged every 3 days) 1 week before muscle injury and was prolonged until euthanasia. SIS3 (Sigma-Aldrich, S0447-5MG; 10 mg kg^−^^1^) in PBS and bortezomib (Teva, TH000345/LF2.3; 0.5 mg kg^−^^1^) in 10% DMSO diluted in PBS were administered i.p. (from 1 to 5 d.p.i.). For long-term treatments, 3-month-old mdx and mdx/p16-3MR mice were administered with D+Q or GCV, respectively, twice a week for 2 months.

### Muscle regeneration

Mice were anaesthetized with ketamine–xylazine (80 and 10 mg kg^−^^1^ respectively; i.p.) or isoflurane. Regeneration of skeletal muscle was induced by intramuscular injection of CTX (Latoxan, L8102; 10 µM) as described previously^51^. At the indicated times after injury, the mice were euthanized and the muscles were dissected, frozen in liquid-nitrogen-cooled isopentane, and stored at −80 °C until analysis.

### Heterografting

Heterografting experiments were performed according to the protocol described previously^52^. In brief, the EDL muscle was removed from the anatomical bed of either p16-3MR or WT mice and was transplanted onto the surface of the TA muscle of the p16-3MR or WT recipient mouse or vice versa. Muscle grafts were collected on day 7 after transplantation.

### Muscle force measurement

Ex vivo force measurements of EDL muscles were assessed as previously described^53^ using and 300B apparatus (Aurora Scientific). Force was normalized per muscle area, determined by dividing the muscle mass by the product of length and muscle density of (1.06 mg mm^−3^), to calculate the specific force (mN mm^−2^).

### p16-3MR *Renilla* luciferase reporter assay

In vivo, *Renilla* luciferase activity was measured in the TA, quadriceps and gastrocnemius muscles of p16-3MR mice. Anaesthetized mice were injected intramuscularly with coelenterazine H (PerkinElmer, 760506) and luciferase activity was immediately measured using the IVIS Lumina III (PerkinElmer) system. In vitro, *Renilla* luciferase activity was measured from the cryopreserved diaphragm and TA muscles using the Dual-Luciferase Reporter Assay Kit (Promega, E1910). The signal was measured using the luminometer Centro LB 960 (Berthold Technologies) and values were normalized to the total protein extracted measured using Bradford method (Protein Assay, Bio-Rad, 500–0006), and the damaged area was measured after haematoxylin and eosin (H&E) staining.

### Cell isolation by flow cytometry

Muscles were mechanically disaggregated and incubated in Dulbecco’s modified Eagle’s medium (DMEM) containing liberase (Roche, 177246) and dispase (Gibco, 17105-041) at 37 °C with agitation for 1–2 h. When required, SPiDER-β-gal reagent (Dojindo, SG02; 1 µM) was added during the second hour. The supernatant was then filtered and cells were incubated in lysis buffer (BD Pharm Lyse, 555899) for 10 min on ice, resuspended in PBS with 2.5% fetal bovine serum (FBS) and counted. BV711-conjugated anti-CD45 (BD, 563709; 1:200), APC-Cy7-conjugated anti-F4/80 (BioLegend, 123118; 1:200), PE-conjugated anti-α7-integrin (Ablab, AB10STMW215; 1:200), APC-conjugated anti-CD31 (eBioscience, 17-0311-82; 1:200) and PE-Cy7-conjugated anti-SCA1 (BioLegend, 108114; 1:200) antibodies were used to isolate MCs (CD45^+^F4/80^+^), SCs (α7-integrin^+^CD45^−^F4/80^−^CD31^−^) and FAPs (SCA1^+^CD45^−^F4/80^−^α7-integrin^−^CD31^−^). PE-Cy7-conjugated anti-CD45 antibodies (BioLegend, 103114) were used to isolate CD45-positive and CD45-negative populations (Extended Data Fig. 1h). SPiDER-β-gal (SPiDER) was used to isolate senescent cells (SPiDER^+^) from non-senescent cells (SPiDER^−^) of each cell type (Extended Data Figs. 1h and 4a) (Supplementary Table 1). Cells were sorted using the FACS Aria II (BD) system. Cell lineage was confirmed by specific-cell marker expression (Extended Data Fig. 4b–d). Isolated cells were used either for RNA extraction, cell cultures, engraftments, proliferation assays or plated onto glass slides (Thermo Fisher Scientific, 177402) for immunostaining and SA-β-gal analysis.

To isolate ROS^high^ and ROS^low^ populations, the digested muscle was stained with CellRox Green reagent (Invitrogen, C10444; 5 µM) according to the manufacturer’s protocol and PE-Cy7-conjugated anti-CD45 (BioLegend, 103114; 1:200), PE-Cy7-conjugated anti-CD31 (BioLegend, 102418; 1:200), PE-conjugated anti-α7-integrin (Ablab, AB10STMW215; 1:200) and APC-conjugated anti-SCA1 (BioLegend, 108111; 1:200) antibodies to separate SCs (α7-integrin^+^CD45^−^CD31^−^) and FAPs (SCA1^+^CD45^−^α7-integrin^−^CD31^−^) (Supplementary Table 1). CellRox^high^ and CellRox^low^ cells were sorted using the FACS Aria II (BD) system. Isolated cells were used for cell cultures and proliferation assays. The acquisition was performed using the BD FACS Diva software.

### Senescent cell transplantation

Cells transplants were performed as described previously^5^, following an adapted protocol^54^. FACS-isolated SPiDER^+^ and SPiDER^−^ cells were collected, resuspended in 20% FBS DMEM medium, labelled with Vybrant Dil Cell Labelling solution (Invitrogen, V22889) according to manufacturer instructions and injected into the TA muscles of recipient mice that were either uninjured or previously injured using the freeze crush method 2 days before^55^. The cell-type proportions of MCs, SCs and FAPs were controlled in the transplanted SPiDER^+^ and SPiDER^−^ populations. Each TA muscle was engrafted with 10,000 cells, except when each senescent cell type was transplanted separately (Fig. 5k), where 5,000 cells were engrafted. Engrafted muscles were collected and processed for muscle histology 4 days after cell transplantation.

### RNA interference

Freshly sorted cells or C2C12 cells (ATCC, CRL-1772) were transfected with siRNA targeting *Cd36* (On-Target plus SmartPool, Dharmacon, L-062017-00-0005; 5 nM) or unrelated sequence as control (On-Target plus non-targeting siRNA Pool, Dharmacon, D-001810-10-05; 5 nM) using the DharmaFect protocol (Dharmacon, T-2003-02). Target sequences for *Cd36* siRNA were as follows: 5′-CCACAUAUCUACCAAAAUU-3′, 5′-GAAAGGAUAACAUAAGCAA-3′, 5’-AUACAGAGUUCGUUAUCUA-3’, 5’-GGAUUGGAGUGGUGAUGUU-3’. Freshly sorted cells after incubation with siRNAs for 3 hours were washed and engrafted.

### Cytokine array

Cytokine antibody arrays (R&D Systems, ARY028; Abcam, ab193659) were used according to the manufacturer’s protocol. For cells, freshly sorted cells were cultured for 24 h in serum-free DMEM. Cell culture supernatants were collected, centrifuged and incubated with the membranes precoated with captured antibodies. For tissue interstitial fluid, skeletal muscles of mice were dissected and slowly injected with a PBS solution with a Complete Mini EDTA-free protease inhibitor cocktail (Roche, 11836170001). The PBS exudate was then recovered centrifuged and incubated with the membranes precoated with captured antibodies. The membranes were then incubated with detection antibodies, streptavidin–HRP and Chemi Reagent Mix. The immunoblot images were captured and visualized using the ChemiDoc MP Imaging System (Bio-Rad) and the intensity of each spot in the captured images was analysed using the publicly available ImageJ software.

### Proliferation assays

To assess proliferation in vivo, muscles were injured by local CTX injection, and the mice were administered with ethynyl-labelled deoxyuridine (EdU, Invitrogen, A10044; 25.5 mg kg^−^^1^; i.p.) 2 h before euthanasia at 4 d.p.i. The muscles were collected and processed for immunofluorescence staining in tissue slides or cell isolation by FACS. EdU-labelled cells were detected using the Click-iT EdU Imaging Kit (Invitrogen, C10086). EdU-positive cells were quantified as the percentage of the total number of cells analysed. In vitro proliferation was quantified on freshly sorted SCs, seeded in 20% FBS Ham’s F10 medium supplemented with b-FGF (Peprotech, 100-18B-250UG; 2.5 ng ml^−^^1^) in collagen-coated plates. After 3 days of culture, SCs were pulse-labelled with bromodeoxyuridine (BrdU, Sigma-Aldrich, B9285-1G; 1.5 μg ml^−^^1^) for 1 h. BrdU-labelled cells were detected by immunostaining using rat anti-BrdU antibodies (Abcam, AB6326, 1:500) and a specific secondary biotinylated donkey anti-rat antibody (Jackson Immunoresearch, 712-066-150, 1:250). Antibody binding was visualized using Vectastain Elite ABC reagent (Vector Laboratories, PK-6100) and 3,3′-diaminobenzidine. BrdU-positive cells were quantified as the percentage of the total number of cells analysed.

### Transwell assay

SCs were freshly isolated from regenerating muscle tissue at 3 d.p.i. and plated onto 24-well plates (Falcon, 353047) in 20% FBS DMEM supplemented with b-FGF. Subsequently, medium or freshly sorted SPiDER^+^ and SPiDER^−^ cell populations (Fig. 5i) or etoposide-induced senescent C2C12 cells (Fig. 6i) were seeded on a 0.4-µm-pore-size cell culture insert (Falcon, 353495) using the same medium. After 3 days of culture, a proliferation assay was performed on SCs with BrdU labelling as described above.

### In vitro treatments

ROS^high^ and ROS^low^ SCs and FAPs were freshly isolated from regenerating muscle at 24 h after injury, seeded and cultured in the presence of NAC (10 mM) or vehicle for 3 days. After the treatment, cells were fixed and further processed for staining. C2C12 cells maintained in 10% FBS DMEM were treated with etoposide (Sigma-Aldrich, E1383, 1 µM) for 5 days to induce senescence and were collected for RNA extraction and RT–qPCR. Cells were stained with a β-galactosidase staining kit (as described below) to confirm their senescent state.

### Cell staining

SA-β-galactosidase (SA-β-gal) activity was detected in freshly sorted cells and cell cultures using the senescence β-galactosidase staining kit (Cell signalling, 9860) according to the manufacturer’s instructions. Lipid droplets were stained with Oil Red O (Sigma-Aldrich, O0625) according to manufacturer instructions. ROS levels were measured by immunofluorescence using CellRox Green reagent (Invitrogen, C10444; 5 µM) according to the instructions. TUNEL assays were performed using the In Situ Cell Death Detection Kit, Fluorescein (Roche, 11684795910), cells treated with DNase were used as a positive control of the staining according to the manufacturer’s description.

### Muscle histology, immunofluorescence and immuno-FISH

Muscles were embedded in OCT solution (TissueTek, 4583), frozen in isopentane cooled with liquid nitrogen and stored at −80 °C until analysis. Muscle cryosections (thickness, 10 μm) were collected and stained for SA-β-gal (AppliChem, A1007,0001), H&E (Sigma-Aldrich, HHS80 and 45235), MYH3 (DSHB, F1.652), Sirius Red (Sigma-Aldrich, 365548) or used for immunofluorescence (Supplementary Table 1). The CSA of H&E- and MYH3-antibody-stained sections, the percentage of muscle area positive for Sirius Red staining and the number of SA-β-gal^+^ cells were quantified using Image J. Double immunofluorescence was performed by the sequential addition of each primary and secondary antibody using positive and negative controls. The sections were air-dried, fixed, washed on PBS and incubated with primary antibodies according to the standard protocol after blocking with a high-protein-containing solution in PBS for 1 h at room temperature. Subsequently, the slides were washed with PBS and incubated with the appropriate secondary antibodies and labelling dyes. Telomere immuno-FISH was performed after γH2AX immunofluorescence staining with telomeric PNA probe (Panagene, F1002-5) as described previously^56^.

### Digital image acquisition

Digital images were acquired using an upright DMR6000B microscope (Leica) with a DFC550 camera for immunohistochemical colour pictures; a Thunder imager 3D live-cell microscope (Leica Microsystems) with hardware autofocus control and a Leica DFC9000 GTC sCMOS camera, using HC PL FLUOTAR ×10/0.32 PH1 ∞/0.17/ON257C and HC PL FLUOTAR ×20/0.4 CORR PH1 ∞/0-2/ON25/C objectives; a Zeiss Cell Observer HS with a ×20 and x40 air objective and a Zeiss AxioCam MrX camera; and a Leica SP5 confocal laser-scanning microscope with HCX PL Fluotar ×40/0.75 and ×63/0.75 objectives. The different fluorophores (three or four) were excited using the 405, 488, 568 and 633 nm excitation lines. The acquisition was performed using the Leica Application (v.3.0) or LAS X (v.1.0) software (Leica) or Zeiss LSM software Zen 2 Blue.

### RNA isolation and RT–qPCR

Total RNA was isolated from snap-frozen muscles using the miRNAeasy Mini Kit (Qiagen, 1038703). PicoPure (Thermo Fisher Scientific, KIT0204) was used for RNA isolation from sorted cells. For RT–qPCR experiments, DNase digestion of 10 mg of RNA was performed using 2 U DNase (Qiagen, 1010395). cDNA was synthesized from total RNA using SuperScript III Reverse Transcriptase (Invitrogen, 18080-044). For gene expression analysis in freshly sorted SCs, FAPs and MCs, cDNA was pre-amplified using the SsoAdvanced PreAmp Supermix (Bio-Rad, 172-5160) according to the manufacturer’s instructions. qPCR reactions were performed as described previously^57^. Reactions were run in triplicate, and automatically detected threshold cycle values were compared between samples. Transcripts of the *Rpl7* housekeeping gene were used as the endogenous control, with each unknown sample normalized to *Rpl7* content (a list of the primers used in this study is provided in Supplementary Table 2).

### RNA-seq sample and library preparation

Sequencing libraries were prepared directly from the lysed cells, without a previous RNA-extraction step. RNA reverse transcription and cDNA amplification were performed using the SMART-Seq v4 Ultra Low Input RNA Kit for Sequencing from Clontech Takara. The Illumina Nextera XT kit was used for preparing the libraries from the amplified cDNA. Libraries were sequenced using the Illumina HiSeq 2500 sequencer (51 bp read length, single-end, around 20 million reads).

### Bulk RNA-seq data preprocessing

Sequencing reads were preprocessed using the nf-core/rnaseq (v.1.2) pipeline^58^. Read quality was assessed using FastQC (v.0.11.8)^59^. Trim Galore (v.0.5.0)^60^ was used to trim sequencing reads, eliminating the remains of Illumina adaptors and discarding reads that were shorter than 20 bp. The resulting reads were mapped onto the mouse genome (GRCm38, Ensembl^61^ release 81) using HiSAT2 (v.2.1.0)^62^ and quantified using featureCounts (v.1.6.2)^63^. Reads per kilobase per million mapped reads (RPKM) and transcripts per million (TPM) gene expression values were calculated from the trimmed mean of *M*-values (TMM)-normalized counts per million (CPM) values using the Bioconductor package edgeR (v.3.30.0)^64^ and R (v.4.0.0)^65^. Differential gene expression analysis and PCA were performed using the Bioconductor package DESeq2 (v.1.28.1)^66^. Variance-stabilizing transformation of count data was applied to visualize the sample-to-sample distances in PCA. Genes were considered to be differentially expressed if showed an adjusted *P* < 0.05.

### Functional profiling of cell subpopulations

Functional enrichment analysis of the subsets of differentially expressed genes was performed using g:Profiler web server^67^ with the g:SCS significance threshold, ‘Only annotated’ statistical domain scope, and canonical pathway KEGG^68^, Reactome^69^ and Wiki Pathways^70^ sets. For each gene subset, the top five significant gene sets were selected for representation.

### GSEA

The RPKM matrix after the removal of low-count genes (edgeR (v.3.30.0)^64^) was used as an input for the GSEA (v.4.0.3) software^71^. We used the signal-to-noise metric to rank the genes, 1,000 permutations with the gene set permutation type and weighted enrichment statistics. Gene set sizes were chosen as 15–500 for MSigDB 7.0 GO:BP and 10–1,000 for MSigDB 7.0 canonical pathways (BioCarta, KEGG, PID, Reactome and WikiPathways)^72^. Gene sets passing the FDR < 0.25 threshold were processed for further analysis. Network representation and clustering of GSEA results were performed using EnrichmentMap (v.3.2.1)^73^ and AutoAnnotate (v.1.3.2)^74^ for Cytoscape (v.3.7.2)^75^ with the Jaccard coefficient set to 0.25.

### Functional profiling of SASP

We checked whether upregulated genes (DESeq2 adjusted *P* < 0.05 and log_2_[fold change] > 0) from each Sen versus NSen comparison can be expressed in a form of secreted proteins by combining the evidence from multiple data sources: GO^76^ cellular component (GO:CC), Uniprot^77^, VerSeDa^78^, Human Protein Atlas^79^ and experimental data reporting SASP^10,80^. The genes encoding extracellular (GO:CC) and/or secreted (other sources) products, with evidence from at least one source, were included in the final list of SASP genes (1,912 in total). Functional enrichment analysis was performed using the g:Profiler web server^67^ with the g:SCS significance threshold, ‘Only annotated’ statistical domain scope, and canonical pathway sets from KEGG, Reactome and Wiki Pathways. Gene sets passing the FDR < 0.05 threshold were processed for further analysis. Network representation and clustering of the g:Profiler results were performed using EnrichmentMap (v.3.2.1) and AutoAnnotate (v.1.3.2) for Cytoscape (v.3.7.2) with the Jaccard coefficient set to 0.25.

### Comparative enrichment analysis of senescent cells and previously published ageing datasets

We used the minimum hypergeometric test implemented in the R package mHG (v.1.1)^81^ for the comparative enrichment analysis of senescent cells and previously published ageing datasets: mouse^36^, rat (Gene Expression Omnibus (GEO): GSE53960), African turquoise killifish (GEO: GSE69122), and human (GTEx^82^ v6p). Data processing and analysis were performed as described previously^36^.

### scRNA-seq and analysis

scRNA-seq was performed using the Chromium Single Cell 3′ GEM, Library & Gel Bead Kit v3, 16 rxns (10x Genomics, PN-1000075) according to the manufacturer’s instructions and targeting a recovery of 5,000 cells per dataset. Each dataset was obtained with a sample size of two mouse biological replicates. The libraries were constructed as instructed in the manufacturer’s protocol and sequenced using the MGI DNBSEQ-T7 sequencer platform. The average read depth across the samples was 15,551 per cell. Sequencing reads were processed with STARsolo (v.2.7.3a)^83^ using the mouse reference genome mm10 (GENCODE vM23 (ref. ^84^)).

From the filtered barcode and count matricesm, downstream analysis was performed using R (v.4.0.3). Quality control, filtering, data clustering, visualization and differential expression analysis were performed using the Seurat (v.4.0.3) and DoubletFinder (v.2.0) R packages^85,86^. Datasets were processed following Seurat standard integration protocol according to the tutorial instructions. Genes expressed in less than 3 cells and cells with fewer than 500 features, less than 2,000 transcripts and more than 20% reads mapping to mitochondrial genes as well as cells identified as doublets by DoubletFinder were removed. PCA was performed for dimensionality reduction and the first 30 components were used for UMAP embedding and clustering.

### ATAC-seq sample and library preparation

Omni-ATAC-seq was performed in freshly sorted cells as described previously^87,88^. After the transposition reaction and purification, the transposed fragments were amplified using 50 μl of PCR mix (20 µl of DNA, 2.5 µl of custom Nextera PCR primers 1 and 2, and 25 µl of KAPA HiFi HS Ready Mix for a total of 15 cycles). The PCR amplification conditions were as follows: 72 °C for 5 min; 95 °C for 30 s; 15 cycles of 95 °C for 10 s, 63 °C for 30 s and 72 °C for 60 s; and a final extension at 72 °C for 5 min. After PCR amplification, the libraries were purified, and the size was selected from 150 to 800 bp using AMPure XP beads. Paired-end sequencing was performed with 50 cycles on the Illumina NovaSeq 6000 platform.

### Bulk ATAC-seq data preprocessing

Read quality was assessed using FastQC (v.0.11.8). All adaptors were removed using Fastp (v.0.21.0)^89^. The clean reads were then aligned to mm10 mouse genome assembly using Bowtie2 (v.2.2.5)^90^ with the settings ‘--very sensitive’. Low-mapping-quality reads were removed using samtools (v.1.3.1)^91^ with the settings ‘-q 30’. BigWig files were generated using deeptools (v.3.3.1)^92^ with the settings ‘-normalizeUsing CPM’. Peaks were called using Macs2 (v.2.1.0)^93^ with the options ‘--nomodel --keep-dup -q 0.01’. For differential accessibility analysis, union peak sets were created using Bedtools (v.2.29.2)^94^, reads corresponding to each region were assigned by FeatureCounts. Differentially accessible peaks were identified using DESeq2 (v.1.24.0) with the criteria of adjusted *P* < 0.1 and an absolute value of log_2_[fold change] > 1. Differentially accessible peaks were further annotated by HOMER (v.4.10.4)^95^, the associated motif enrichment analysis was performed by HOMER using the default settings.

### Analysis of senescence-induced changes in promoter chromatin accessibility

An *MA* plot (log_2_-transformed fold change versus mean average) was used to visualize changes in chromatin accessibility for all peaks. As a peak score, we used an average of TPM-normalized read counts: (1) reads per kilobase were calculated by division of the read counts by the length of each peak in kilobases; (2) the per million scaling factor was calculated as a sum of all reads per kilobase for each sample; (3) reads per kilobase were divided by the per million scaling factor; (4) peaks with the ‘promoter-TSS’ annotation TSS ± 1kb were selected and the average was calculated for each group. For *MA* plots, we included only those peaks with an average normalized signal > 5. The number of peaks with a log_2_-transformed fold change of >1 or <−1 was calculated. Normalized ATAC-seq signal profiles of proximal promoters were visualized for key genes using the Integrative Genomic Viewer (v.2.8.13)^96^.

### Transcription factor analysis and activity prediction

For the analysis of transcription regulation, we combined the results of several methods: (1) motif enrichment analysis of differentially expressed genes with the TRANSFAC_and_JASPAR_PWMs and ENCODE_and_ChEA_Consensus_TFs_from_ChIP-X libraries using the R package EnrichR (v.2.1)^97^; (2) upstream regulator analysis of differentially expressed genes using the commercial Ingenuity Pathway Analysis (IPA, QIAGEN) software^98^; (3) analysis of transcription factor differential expression using DESeq2 (v.1.28.1); (4) motif enrichment analysis of differentially accessible regions using HOMER (v.4.10.4).

Potential regulators from EnrichR and IPA results passing the threshold of *P* < 0.05 were used to build a union set of transcription factors, which was further filtered to retain only the molecules with DESeq2 baseMean value > 0. For further validation of the activity status, transcription factors were matched to the known HOMER motifs passing the Benjamini *Q* < 0.05 threshold.

A discrete scoring scale (inhibited, possibly inhibited, unknown/contradictory, possibly activated, activated) was used to evaluate transcription factor activity based on combined evidence from the EnrichR, IPA, DESeq2 and HOMER results. We used *z*-score statistics to define the activity status of transcription factors from the EnrichR analysis results by matching the differential expression of target genes with activatory and inhibitory interactions from the Bioconductor package DoRothEA (v.1.0.0)^99^ and the web-based TRRUST v.2 database^100^. To define the activity status of transcription factors from IPA upstream regulators analysis results, IPA-calculated *z*-score and analysis bias was taken into account. Activity predictions were further corrected by differential expression of transcription factors using DESeq2. The expression *z*-score statistical value was calculated to functionally classify transcription factors as activators or repressors on the basis of the proportion of upregulated and downregulated target genes. We further calculated the chromatin accessibility *z*-score to estimate the prevalence of HOMER motif enrichment in open versus closed regions that together with the predicted transcription factor function enabled us to validate the RNA-seq activity predictions using ATAC-seq data.

To estimate the level of confidence, for each enrichment result, we calculated a discrete ‘trust’ score, with each point assigned for: (1) EnrichR adjusted *P* < 0.05; (2) IPA *P* < 0.05; (3) activity status ‘activated’ or ‘inhibited’; (4) unidirectional absolute *z*-scores of >2 from both the EnrichR and IPA results; (5) concordance between transcription factor differential expression and the prediction of its activity score; (6) activity validated by the analysis of ATAC-seq data. Transcription factors with average trust > 1 were processed for further analysis.

### Functional profiling of transcription factor target gene regulation

For each transcription factor, we merged the target genes from EnrichR and IPA results, split them into upregulated and downregulated and processed them to functional enrichment analysis of canonical pathways (KEGG, Reactome) and GO:BP using R package gprofiler2 (v.0.1.9)^101^ with the following parameters: correction method ‘FDR’, ‘custom_annotated’ domain score consisting of target genes for all studied transcription factors. Electronic GO annotations were excluded. Gene sets that passed the FDR < 0.05 threshold were processed for further analysis. For GO:BP, we selected the gene sets with a term size of >15 and <500 genes. Transcription factors were further mapped based on the matching terms from the gprofiler2 results to the main functional clusters of the gene sets created previously in GSEA/Cytoscape analysis.

Transcription factors mapped to the same functional cluster in ≥8 (out of 12) Sen versus NSen comparisons were processed for further filtering. We scored as 1 point in each case when any of the following attributes had a value above the upper quartile for a given cluster: number of comparisons, percentage of GSEA terms among all terms with enrichment, −log_10_ of the average minimum FDR and average trust score. Moreover, we scored as 1 point if the transcription factor was associated with senescence in literature. For graphical representation, we selected examples of transcription factors and target genes based on literature research: 19 transcription factors (out of 29 with a score of ≥2) mapped to 9 clusters (matrix remodelling/fibrosis, interferon signalling, chemotaxis, lipid uptake, IGF regulation, detoxification, gene expression and protein translation, cell cycle, and DNA repair).

### Functional profiling of transcriptional regulation of SASP

For each transcription factor upregulated, target genes from the EnrichR and IPA results were merged and intersected with the list of SASP genes. For SASP genes, we extracted GO:MF terms, clustered them into 12 categories (adhesion molecule, chemokine, complement component, cytokine, enzyme, enzyme regulator, extracellular matrix constituent, growth factor, hormone, ligand, proteinase and receptor) and estimated the enrichment of GO:MF clusters with a hypergeometric test using the R function phyper. Correction for multiple comparisons was performed using the Benjamini–Hochberg procedure.

Transcription factors that had target enrichment in the same GO:MF cluster with *P* < 0.05 in ≥8 (out of 12) Sen versus NSen comparisons were processed for further filtering. We scored 1 point in each case in which any of the following attributes had a value above the upper quartile for a given cluster: the percentage of secreted proteins among targets, the number of comparisons with *P* < 0.05, the number of comparisons with adjusted *P* < 0.05, −log_10_ of the average *P* value, the average trust score. Moreover, we used ATAC-seq data analysis to score 1 point in cases in which the transcription factor motif was present in the promoter region of at least one SASP gene within the cluster. For graphical representation, we selected 17 transcription factors with a score of ≥2 and with ≥3 comparisons with adjusted *P* < 0.05. They were associated with five GO:MF categories (extracellular matrix constituent, cytokine, chemokine, complement component and growth factor), for which we selected the most common target genes.

### Analysis of lipid metabolism gene set

For the analysis of lipid metabolism, we constructed a gene set using data from multiple sources: KEGG pathway maps (fatty acid degradation, cholesterol metabolism, regulation of lipolysis in adipocytes), WikiPathways (fatty acid oxidation, fatty acid beta oxidation, mitochondrial LC-fatty acid beta-oxidation, fatty acid omega oxidation, fatty acid biosynthesis, triacylglyceride synthesis, sphingolipid metabolism (general overview), sphingolipid metabolism (integrated pathway), cholesterol metabolism (includes both Bloch and Kandutsch–Russell pathways) and cholesterol biosynthesis) and literature research^102–104^. We further estimated the expression of these genes by filtering DESeq2 results (adjusted *P* < 0.05 in at least 3 out of 12 comparisons) and extracted log_2_-transformed fold change values to plot the difference in expression between senescent and non-senescent cells.

### Reconstruction of ligand–receptor mediated cell–cell communication networks

For reconstructing cell–cell communication networks, we modified the single-cell-based method, FunRes, to account for bulk gene expression profiles^39^. In brief, transcription factors with an expression value of more than 1 TPM were considered to be expressed. Receptors regulating these transcription factors were detected using a Markov chain model of signal transduction to detect high-probability intermediate signalling molecules^105^. Ligand–receptor interactions between two cell populations were reconstructed if (1) the receptor is expressed and regulates any transcription factor, (2) the ligand is expressed and (3) the receptor–ligand interaction is contained in the cell–cell interaction scaffold. Finally, a score is assigned to every interaction by multiplying the average receptor and ligand expression in their respective cell populations. Significance was assessed by permuting cell population labels 100 times and recomputing the interaction scores in the permuted datasets. Interactions were considered to be significant if they were at least 2 s.d. greater than the mean of the permuted interaction scores. Only significant interactions were retained in the final network.

### Downstream analysis of senescence-induced ligand–receptor interactions

For the functional profiling, we selected ligand–receptor interactions between three senescent cell populations (SCs, FAPs and MCs) and a non-senescent SC population in old mice at 3 d.p.i. We used the Bioconductor package SPIA (v.2.40.0)^40^ with a reduced set of non-disease KEGG pathway maps to evaluate the activity of a pathway’s downstream ligand–receptor interactions. For each interaction, differentially expressed target transcription factors in non-senescent SCs were split into upregulated and downregulated in comparison to senescent SCs. As a reference set of genes, we took a list of target transcription factors from all of the interactions studied. SPIA analysis was performed with 2,000 permutations, and pPERT and pNDE were combined using the Fisher’s product method. Pathways passing the pGFdr < 0.05 threshold were considered to be significantly enriched. For each pathway, we calculated the ratio of ligand–receptor interactions that activate or inhibit the pathway to the total number of interactions analysed. For results representation, we selected eight activated and eight inhibited pathways with the highest ratio of interactions.

### Statistical analysis

The sample size of each experimental group is described in the corresponding figure caption, and all of the experiments were conducted with at least three biological replicates unless otherwise indicated. GraphPad Prism was used for all statistical analyses except for sequencing-data analysis. Quantitative data displayed as histograms are expressed as mean ± s.e.m. (represented as error bars). Results from each group were averaged and used to calculate descriptive statistics. Mann–Whitney *U*-tests (independent samples, two-tailed) were used for comparisons between groups unless otherwise indicated. *P* < 0.05 was considered to be statistically significant. Experiments were not randomized.

### Reporting summary

Further information on research design is available in the Nature Portfolio Reporting Summary linked to this article.

## Online content

Any methods, additional references, Nature Portfolio reporting summaries, source data, extended data, supplementary information, acknowledgements, peer review information; details of author contributions and competing interests; and statements of data and code availability are available at 10.1038/s41586-022-05535-x.

## Supplementary information

Supplementary Information Supplementary Tables 1 and 2.

Reporting Summary

## Data Availability

The bulk RNA-seq, scRNA-seq and ATAC-seq data supporting the findings of this study have been deposited at the GEO under accession number GSE196613. Source data are provided with this paper.

## Source data

Source Data Fig. 1
Source Data Fig. 2
Source Data Fig. 3
Source Data Fig. 4
Source Data Fig. 5
Source Data Fig. 6
Source Data Extended Data Fig. 1
Source Data Extended Data Fig. 2
Source Data Extended Data Fig. 3
Source Data Extended Data Fig. 4
Source Data Extended Data Fig. 5
Source Data Extended Data Fig. 6
Source Data Extended Data Fig. 7
Source Data Extended Data Fig. 8
Source Data Extended Data Fig. 9
Source Data Extended Data Fig. 10
Source Data Extended Data Fig. 11
Source Data Extended Data Fig. 12
