# Author Correction: Senescence atlas reveals an aged-like inflamed niche that blunts muscle regeneration

**DOI:** 10.1038/s41586-023-05765-7

**Published:** 2023-02-06

**Authors:** Victoria Moiseeva, Andrés Cisneros, Valentina Sica, Oleg Deryagin, Yiwei Lai, Sascha Jung, Eva Andrés, Juan An, Jessica Segalés, Laura Ortet, Vera Lukesova, Giacomo Volpe, Alberto Benguria, Ana Dopazo, Salvador Aznar Benitah, Yasuteru Urano, Antonio del Sol, Miguel A. Esteban, Yasuyuki Ohkawa, Antonio L. Serrano, Eusebio Perdiguero, Pura Muñoz-Cánoves

**Affiliations:** 1 Department of Medicine and Life Sciences, Pompeu Fabra University, Barcelona, Spain; 2 CIBERNED, Barcelona, Spain; 3 Laboratory of Integrative Biology, Guangzhou Institutes of Biomedicine and Health, Chinese Academy of Sciences, Guangzhou, China; 4 Key Laboratory of Regenerative Biology, Guangzhou Institutes of Biomedicine and Health, Chinese Academy of Sciences, Guangzhou, China; 5 Guangdong Provincial Key Laboratory of Stem Cells and Regenerative Medicine, Guangzhou Institutes of Biomedicine and Health, Chinese Academy of Sciences, Guangzhou, China; 6 CIC bioGUNE-BRTA (Basque Research and Technology Alliance), Bizkaia Technology Park, Derio, Spain; 7 University of Science and Technology of China, Hefei, China; 8 Genomic Unit, Centro Nacional de Investigaciones Cardiovasculares and CIBERCV, Madrid, Spain; 9 ICREA, Barcelona, Spain; 10 Institute for Research in Biomedicine and BIST, Barcelona, Spain; 11 Laboratory of Chemistry & Biology, Graduate School of Pharmaceutical Sciences and School of Medicine, The University of Tokyo, Tokyo, Japan; 12 Computational Biology Group, Luxembourg Centre for Systems Biomedicine, University of Luxembourg, Esch-sur-Alzette, Luxembourg; 13 IKERBASQUE, Basque Foundation for Science, Bilbao, Spain; 14 Bioland Laboratory, Guangzhou Regenerative Medicine and Health Guangdong Laboratory, Guangzhou, China; 15 Division of Transcriptomics. Medical Institute of Bioregulation, Kyushu University, Fukuoka, Japan; 16 Altos labs Inc, San Diego, CA USA; 17 Cardiovascular Regeneration Program, CNIC Centro Nacional de Investigaciones Cardiovasculares, Madrid, Spain

**Keywords:** Senescence, Regeneration

Correction to: *Nature* 10.1038/s41586-022-05535-x Published online 21 December 2022

In the version of this article initially published, the middle name and surname of Salvador Aznar Benitah were wrongly hyphenated, reading as “Aznar-Benitah”. The error has been corrected in the HTML and PDF versions of the article.

