# Supplementary material for: Senescence atlas reveals an aged-like inflamed niche that blunts muscle regeneration
Source: Nature. 2022 Dec 21;613(7942):169–78. doi: 10.1038/s41586-022-05535-x (PMC9812788; doi:10.1038/s41586-022-05535-x)
Supplement: Supplementary file 1 — Supplementary Tables 1 and 2. [file 41586_2022_5535_MOESM1_ESM.pdf]

nature portfolio

https://doi.org/10.1038/s41586-022-05535-x

Supplementary information

Senescence atlas reveals an aged-like inflamed niche that blunts muscle regeneration

In the format provided by the authors and unedited

Antibody	Brand	Reference	Dilution
nGFP	Invitrogen	#A6455	1/400
eMHC	DSHB	#F1.652	Ready to use
p16^{INKA4}	Invitrogen	#MA5-17142	1/100
TCF4	Cell Signaling	#2569S	1/80
CD11b	eBioscience	#14-0112-85	1/100
Lamin B1	Abcam	#ab16048-100	1/100
CD36	Invitrogen	#MA5-14112	1/100
ɣH2AX	Cell Signaling	#2577S	1/50
Pax7	Abcam	#ab34360	1/100
Pax7	Santa Cruz	#sc-81648	1/20
Ki67	Abcam	#ab15580	1/100
PDGF Receptor alpha (D1E1E) XP	Cell signaling	#3174	1/200
F4/80	Abcam	#ab6640	1/200
PDGF Receptor alpha	Mybiosource/Bionova	#MBS9700557	1/100
p16^{INK4a} (1E12E10)	Fisher Scientific	#MA5-17142	1/100
Human PDGFR alpha	R&D Systems	#AF-307-NA	1/100
Human 53BP1	Abcm	#ab21083	1/100
Human Pax7	Santa Cruz	#sc-81648	1/50
Human CD68	DAKO	#M0718	1/50

Table 1. Antibodies used in this study.

Gene	Forward primer	Reverse primer
p16^{INKA}	CATCTGGAGCAGCATGGAGTC	ATCATCATCACCTGAATCGGGG
mRFP	GACCTCGGCGTCGTAGTG	AAGGGCGAGATCAAGATGAG
p19^{ARF}	TGAGGCTAGAGAGGATCTTGAGA	GCAGAAGAGCTGCTACGTGAA
p21	CCAGGCCAAGATGGTGTCTT	TGAGAAAGGATCAGCCATTGC
Il-6	GGTGACAACCACGGCCTTCCC	AAGCCTCCGACTTGTGAAGTGGT
Il-1b	CCAAAATACCTGTGGCCTTGG	GCTTGTGCTCTGCTTGTGAG
PAI-1	CCGATGGGCTCGAGTATGA	TTGTCTGATGAGTTCAGCATCCA
TNFα	CGCTCTTCTGTCTACTGAACTT	GATGAGAGGGAGGCCATT
IFNɣ	AGCGGCTGACTGAACTCAGATTGTAG	GTCACAGTTTTCAGCTGTATAGGG
Il-12	TCCAGCGCAAGAAAGAAAA	AATAGCGATCCTGAGCTTGC
Ccl12	CCCACTCACCTGCTGCTACT	TCTGGACCCATTCCTTCTTG
Il-18	CTGGCTGTGACCCTCTCTGT	ATCTTCCTTTTGGCAAGCAA
Rpl7	GAAGCTCATCTATGAGAAGGC	AAGACGAAGGAGCTGCAGAAC
Ccl2	CACTCACCTGCTGCTACTCA	GAGCTTGGTGACAAAAACTACAGC
Ccl7	GTCCCCGGGAAGCTGTAATC	GCTTTGGAGTTTGGGTTTTCTT
Ccl8	ACGCTAGCCTTCACTCCAAA	GTGACTGGAGCCTTATCTGG
Apoe	CTCCCAAGTCACACAAGAACTG	CCAGCTCCTTTTTGTAAGCCTTT
Col3a1	TGACTGTCCCACGTAAGCAC	GAGGGCCATAGCTGAACTGA
Col6a3	CCAACAGCATGGAGTCATGG	GCATTGAAGTTGGATGGCCC
Timp2	TGCAATGCAGACGTAGTGAT	ATAGATGTCATTCCCGGAAT
Igfbp4	TGAGAGCGAACATCCCAACAA	TGTCCCCACGATCTTCATCTT
Cxcl10	TGCCCACGTGTTGAGATCAT	AAGGAGCCCTTTTAGACCTTTT
Igfbp7	TGCGAGCAAGGGTCTCTGAT	GTTGGGATCCCGATGACCTC

Table 2. Primers used for RT-qPCR in this study.
