# Supplementary material for: Senescence atlas reveals an aged-like inflamed niche that blunts muscle regeneration
Source: Nature. 2022 Dec 21;613(7942):169–78. doi: 10.1038/s41586-022-05535-x (PMC9812788; doi:10.1038/s41586-022-05535-x)
Supplement: Supplementary file 2 — Reporting Summary [file 41586_2022_5535_MOESM2_ESM.pdf]

nature portfolio

Corresponding author(s): Pura Munoz-Canoves

Last updated by author(s): 10/25/2022

Reporting Summary

Nature Portfolio wishes to improve the reproducibility of the work that we publish. This form provides structure for consistency and transparency in reporting. For further information on Nature Portfolio policies, see our Editorial Policies and the Editorial Policy Checklist.

Statistics

For all statistical analyses, confirm that the following items are present in the figure legend, table legend, main text, or Methods section.

n/a	Confirmed	
☐	☒	The exact sample size (*n*) for each experimental group/condition, given as a discrete number and unit of measurement
☐	☒	A statement on whether measurements were taken from distinct samples or whether the same sample was measured repeatedly
☐	☒	The statistical test(s) used AND whether they are one- or two-sided<br>*Only common tests should be described solely by name; describe more complex techniques in the Methods section.*
☒	☐	A description of all covariates tested
☐	☒	A description of any assumptions or corrections, such as tests of normality and adjustment for multiple comparisons
☐	☒	A full description of the statistical parameters including central tendency (e.g. means) or other basic estimates (e.g. regression coefficient) AND variation (e.g. standard deviation) or associated estimates of uncertainty (e.g. confidence intervals)
☒	☐	For null hypothesis testing, the test statistic (e.g. *F*, *t*, *r*) with confidence intervals, effect sizes, degrees of freedom and *P* value noted<br>*Give P values as exact values whenever suitable.*
☒	☐	For Bayesian analysis, information on the choice of priors and Markov chain Monte Carlo settings
☒	☐	For hierarchical and complex designs, identification of the appropriate level for tests and full reporting of outcomes
☐	☒	Estimates of effect sizes (e.g. Cohen's *d*, Pearson's *r*), indicating how they were calculated

Our web collection on statistics for biologists contains articles on many of the points above.

Software and code

Policy information about availability of computer code

Data collection	Leica Application software v3.0 or LAS X v1.0 software1219 (Leica) or Zeiss LSM software: "Zen 2 Blue" were used for capturing images at wide field or confocal micrsocopes. BD FACS Diva Software was used for collecting FACS Data.
Data analysis	For statistical analysis GraphPad Prism version 8.0.1 was used. For image analysis, Image J Fiji was used. For FACS analysis, , FlowJo (v10.4) was used.<br>Bulk RNA-seq Data Pre-processing: nf-core/rnaseq 1.2 pipeline; FastQC 0.11.8; Trim Galore 0.5.0; HiSAT2 2.1.0; featureCounts 1.6.2 ; Bioconductor package edgeR 3.30.0; R 4.0.0 ; Bioconductor package DESeq2 1.28.1.<br>Functional profiling: g:Profiler web server; EnrichmentMap 3.2.1; AutoAnnotate 1.3.2 for Cytoscape 3.7.2.<br>Gene Set Enrichment Analysis (GSEA): edgeR 3.30.0; GSEA 4.0.3 software; EnrichmentMap 3.2.1; AutoAnnotate 1.3.2 for Cytoscape 3.7.2.<br>Single-cell RNA-sequencing and analysis: STARsolo 2.7.3a; R 4.0.3.; Seurat 4.0.3; DoubletFinder 2.0 R.<br>Bulk ATAC-seq data pre-processing: FastQC 0.11.8.; Fastp 0.21.0 ; Bowtie2 2.2.5; samtools 1.3.1; deeptools 3.3.1 ; Macs2 2.1.0 ; Bedtools 2.29.2 ; FeatureCounts. ; HOMER 4.10.4.<br>Analysis of senescence-induced changes in promoter chromatin accessibility: Integrative Genomic Viewer 2.8.13.<br>Transcription factor analysis and activity prediction:EnrichR 2.1; QIAGEN's Ingenuity Pathway Analysis (IPA, QIAGEN) ; DESeq2 1.28.1; HOMER 4.10.4.; Bioconductor package DoRothEA 1.0.0.<br>Functional profiling of transcription factor target gene regulation: R package gprofiler2 0.1.9<br>For reconstructing cell-cell communication networks, we used FunRes (Jung et al.,1451 2020).<br>Downstream analysis of senescence-induced ligand-receptor interactions: Bioconductor package SPIA 2.40.0

For manuscripts utilizing custom algorithms or software that are central to the research but not yet described in published literature, software must be made available to editors and reviewers. We strongly encourage code deposition in a community repository (e.g. GitHub). See the Nature Portfolio guidelines for submitting code & software for further information.

Data

Policy information about availability of data

All manuscripts must include a data availability statement. This statement should provide the following information, where applicable:

- Accession codes, unique identifiers, or web links for publicly available datasets
- A description of any restrictions on data availability
- For clinical datasets or third party data, please ensure that the statement adheres to our policy

The scRNAseq, bulk RNA-seq and ATAC-seq data that support the findings of this study have been deposited in GEO under the accession code GSE196613.

Field-specific reporting

Please select the one below that is the best fit for your research. If you are not sure, read the appropriate sections before making your selection.

☒ Life sciences ☐ Behavioural & social sciences ☐ Ecological, evolutionary & environmental sciences

For a reference copy of the document with all sections, see nature.com/documents/nr-reporting-summary-flat.pdf

Life sciences study design

All studies must disclose on these points even when the disclosure is negative.

Sample size	Preliminary experiments were performed to determine the sample size. Sample size sufficiency were determined by previous experiments from our laboratories. No statistical methods were used to predetermine the sample size.
Data exclusions	For aging experiments, the mice determined by the animal facility veterinary team as healthy were used in the experiments. Mice determined as not healthy were excluded, using pre-established criteria for acceptable mouse health status.
Replication	All attempts at replication were successful. Figure legends state how many times each experiment was performed.
Randomization	Mice were randomly allocated to experimental or treatment groups. For experiments other than mice, we did not carry out any randomization because this is either irrelevant or not applicable to these studies.
Blinding	Investigators were not blinded to mouse grouping since animal experiments and assessment of their health status were performed by the same researchers.<br>For other experiments, the investigators were not blinded since analyses relied on unbiased measurements of quantitative parameters. However, standardized procedures for data collection and analysis were used to prevent bias.

Reporting for specific materials, systems and methods

We require information from authors about some types of materials, experimental systems and methods used in many studies. Here, indicate whether each material, system or method listed is relevant to your study. If you are not sure if a list item applies to your research, read the appropriate section before selecting a response.

Materials & experimental systems

n/a	Involved in the study	
☐	☒	Antibodies
☐	☒	Eukaryotic cell lines
☒	☐	Palaeontology and archaeology
☐	☒	Animals and other organisms
☐	☒	Human research participants
☒	☐	Clinical data
☒	☐	Dual use research of concern

Methods

n/a	Involved in the study	
☒	☐	ChIP-seq
☐	☒	Flow cytometry
☒	☐	MRI-based neuroimaging

Antibodies

Antibodies used	Only commercial antibodies have been used.<br><br>BV711-conjugated anti-CD45 (BD, 1103 #563709; 1/200), APC-Cy7-conjugated anti F4/80 (Biolegend, #123118; 1/200), PE-conjugated 1104 anti-α7-integrin (Ablab, #AB10STMW215; 1/200), APC-conjugated anti-CD31 (eBioscience, 1105 #17-0311-82; 1/200) and PE-Cy7-conjugated anti-Sca-1 (Biolegend, #108114; 1/200) antibodies were used to isolate MCs (CD45+ and F4/80+), SCs (α7-integrin+, CD45–, F4/80– 1106 and CD31–) and FAPs (Sca-1+, CD45–, F4/80–, α7-integrin– and CD31–1107). PE-Cy7-conjugated anti-CD45 (Biolegend, #103114; 1/200), PE-Cy7-conjugated anti-CD31 (Biolegend, 1118 #102418; 1/200), PE-conjugated anti-α7-

integrin (Ablab, #AB10STMW215; 1/200), and APC conjugated anti-Sca-1 (Biolegend, #108111; 1/200).
Other antibodies:
nGFP, Invitrogen, #A6455, 1/400
eMHC, DSHB, #F1.652, Ready to use
p16INKA4, Invitrogen, #MA5-17142, 1/100
TCF4, Cell Signaling, #2569S, 1/80
CD11b, eBioscience , #14-0112-85, 1/100
Lamin B1, Abcam, #ab16048-100, 1/100
CD36, Invitrogen, #MA5-14112, 1/100
γH2AX, Cell Signaling, #2577S, 1/50
Pax7, Abcam, #ab34360, 1/100
Pax7, Santa Cruz, #sc-81648, 1/20
Ki67, Abcam, #ab15580, 1/100
PDGF Receptor alpha (D1E1E) XP, Cell signaling, #3174, 1/200
F4/80, Abcam, #ab6640, 1/200
PDGF Receptor alpha , Mybiosource/Bionova, #MBS9700557, 1/100
p16INK4a (1E12E10), Fisher Scientific, #MA5-17142, 1/100
Human PDGFR alpha, R&D Systems, #AF-307-NA, 1/100
Human 53BP1, Abcm, #ab21083, 1/100
Human Pax7, Santa Cruz, #sc-81648, 1/50
Human CD68, DAKO, #M0718, 1/50

Validation

Antibodies were validated by manufacturers or validated in previous studies. Statements on antibody validation are present on the manufacturer's websites along with relevant references. Additional validation was done by the use of negative control (1st ab exclusion) and control tissue samples for IHC/IF, and by the use of negative control (control IgG) and control cells for FACS.

Eukaryotic cell lines

Policy information about cell lines

Cell line source(s)

C2C12 were obtained from ATCC, catalog number CRL-1772

Authentication

Vendor of the commercially-obtained cell lines (ATCC) provide further information on the generation, characteristics and authentication of the cell line in its website. C2C12 cell line was further validated by morphological studies after differentiation of C2C12 myoblasts into myotubes and by PCR/Immunostaining of myoblast/myotube specific genes/proteins.

Mycoplasma contamination

All cell lines were tested for mycoplasma at several times during this research and only used if the results were negative.

Commonly misidentified lines
(See ICLAC register)

The cell line used in this study is not present in the registry of commonly misidentified lines

Animals and other organisms

Policy information about studies involving animals; ARRIVE guidelines recommended for reporting animal research

Laboratory animals

For analysis of wild type mice, C57BL/6J strain was used.
We use the following lines: p16-3MR and DBA/2-mdx
We generated the offspring of intercrossing p16-3MR anad DBA/2-mdx lines..
All experiments were done using age- and weight-matched littermate mice. Both male and female mice were used in each experiment unless stated otherwise. Mice were housed in standard cages under 12-hour light-dark cycles and fed ad libitum with a standard chow diet.
All mice were closely monitored by authors, facility technicians (during treatments) and by an external veterinary scientist responsible for animal welfare.

Wild animals

No wild animals were used in this study.

Field-collected samples

No field-collected samples were used in this study.

Ethics oversight

All experiments followed the principle of the “Three Rs”: replacement, reduction, and refinement according to Directive 63 / 2010 and its implementation in the Member States. All procedures had authorization from the PRBB Animal Research Ethics Committee (PRBB-CEEA) and the local government (Generalitat de Catalunya) and were conducted according to the European Directive 2010/63/EU and Spanish regulations RD 53/2013.

Note that full information on the approval of the study protocol must also be provided in the manuscript.

Human research participants

Policy information about studies involving human research participants

Population characteristics

See above.

Recruitment

Participants were recruited and tested using similar procedures in five sites across Europe belonging to the Myoage

Recruitment	consortium.
Ethics oversight	Human muscle biopsies were obtained via the biobank of the EU/FP7 Myoage Consortium. Ethical approval was received from the local ethics committees at each of the five research centres of the Consortium. All participants provided written informed consent and were medically screened prior to participation.

Note that full information on the approval of the study protocol must also be provided in the manuscript.

Flow Cytometry

Plots

Confirm that:

☒ The axis labels state the marker and fluorochrome used (e.g. CD4-FITC).

☒ The axis scales are clearly visible. Include numbers along axes only for bottom left plot of group (a 'group' is an analysis of identical markers).

☒ All plots are contour plots with outliers or pseudocolor plots.

☒ A numerical value for number of cells or percentage (with statistics) is provided.

Methodology

Sample preparation	Muscles were mechanically disaggregated and incubated in Dulbecco's Modified Eagle's medium (DMEM) containing liberase (Roche, #177246) and dispase (Gibco, #17105-041) at 37°C with agitation for 1-2 hours. When required, SPiDER-β-gal reagent (Dojindo, #SG02; 1 µM) was added during the second hour. The supernatant was then filtered and cells were incubated in lysis buffer (BD Pharm Lyse, #555899) for 10 min on ice, resuspended in PBS with 2.5% fetal bovine serum (FBS), and counted.
Instrument	Cells were sorted using a FACS Aria II (BD).
Software	FACS Diva Software was used for collecting FACS Data. Data was then analyzed using FlowJo software v10.4.
Cell population abundance	Fluorescent microscopic detection, over 95%.
Gating strategy	BV711-conjugated anti-CD45 (BD, #563709; 1/200), APC-Cy7-conjugated anti F4/80 (Biolegend, #123118; 1/200), PE-conjugated anti-α7-integrin (Ablab, #AB10STMW215; 1/200), APC-conjugated anti-CD31 (eBioscience, #17-0311-82; 1/200) and PE-Cy7-conjugated anti-Sca-1 (Biolegend, #108114; 1/200) antibodies were used to isolate MCs (CD45+ and F4/80+), SCs (α7-integrin+, CD45–, F4/80– and CD31–) and FAPs (Sca-1+, CD45–, F4/80–, α7-integrin– and CD31–). PE-Cy7-conjugated anti-CD45 antibody (Biolegend, #103114) was used to isolate CD45 positive and negative populations (see Extended Data Fig. 1f). SPiDER-β-gal (SPiDER) was employed to isolate senescent cells (SPiDER+) from non-senescent cells (SPiDER–) of each cell type

☒ Tick this box to confirm that a figure exemplifying the gating strategy is provided in the Supplementary Information.
